# CCP5 and CCP6 retain CP110 and negatively regulate ciliogenesis

**DOI:** 10.1186/s12915-023-01622-1

**Published:** 2023-05-24

**Authors:** Yujuan Wang, Yuan Zhang, Xinyu Guo, Yiqiang Zheng, Xinjie Zhang, Shanshan Feng, Hui-Yuan Wu

**Affiliations:** 1 School of Pharmaceutical Science and Technology, Tianjin University, 92 Weijin Road, Building 24, Room 417-8, Tianjin, 300072 China; 2 Key Laboratory of Regenerative Medicine, Ministry of Education, Department of Developmental and Regenerative Biology, Jinan University, Guangzhou, 51063 China

**Keywords:** CCP5, CCP6, Polyglutamylation, Ciliogenesis, CP110

## Abstract

**Background:**

The axonemal microtubules of primary cilium undergo a conserved protein posttranslational modification (PTM) — polyglutamylation. This reversible procedure is processed by tubulin tyrosine ligase-like polyglutamylases to form secondary polyglutamate side chains, which are metabolized by the 6-member cytosolic carboxypeptidase (CCP) family. Although polyglutamylation modifying enzymes have been linked to ciliary architecture and motility, it was unknown whether they also play a role in ciliogenesis.

**Results:**

In this study, we found that CCP5 expression is transiently downregulated upon the initiation of ciliogenesis, but recovered after cilia are formed. Overexpression of CCP5 inhibited ciliogenesis, suggesting that a transient downregulation of CCP5 expression is required for ciliation initiation. Interestingly, the inhibitory effect of CCP5 on ciliogenesis does not rely on its enzyme activity. Among other 3 CCP members tested, only CCP6 can similarly suppress ciliogenesis. Using CoIP-MS analysis, we identified a protein that potentially interacts with CCP — CP110, a known negative regulator of ciliogenesis, whose degradation at the distal end of mother centriole permits cilia assembly. We found that both CCP5 and CCP6 can modulate CP110 level. Particularly, CCP5 interacts with CP110 through its N-terminus. Loss of CCP5 or CCP6 led to the disappearance of CP110 at the mother centriole and abnormally increased ciliation in cycling RPE-1 cells. Co-depletion of CCP5 and CCP6 synergized this abnormal ciliation, suggesting their partially overlapped function in suppressing cilia formation in cycling cells. In contrast, co-depletion of the two enzymes did not further increase the length of cilia, although CCP5 and CCP6 differentially regulate polyglutamate side-chain length of ciliary axoneme and both contribute to limiting cilia length, suggesting that they may share a common pathway in cilia length control. Through inducing the overexpression of CCP5 or CCP6 at different stages of ciliogenesis, we further demonstrated that CCP5 or CCP6 inhibited cilia formation before ciliogenesis, while shortened the length of cilia after cilia formation.

**Conclusion:**

These findings reveal the dual role of CCP5 and CCP6. In addition to regulating cilia length, they also retain CP110 level to suppress cilia formation in cycling cells, pointing to a novel regulatory mechanism for ciliogenesis mediated by demodifying enzymes of a conserved ciliary PTM, polyglutamylation.

**Supplementary Information:**

The online version contains supplementary material available at 10.1186/s12915-023-01622-1.

## Background

Primary cilium is a single hair-like microtubule-based structure that protrudes from the cell surface and serves as an “antenna” for cells to sense and transduce outside signals [1]. The disrupted functions of cilia can cause a broad spectrum of degenerative and developmental anomalies, such as kidney dysfunction, photoreceptor degeneration, and situs inversus, collectively referred to as ciliopathy [2]. Primary cilia are usually formed in quiescent cells when centrioles are free from cell division and transformed into basal bodies to support cilia assembly, while cilia disassembly occurs upon cell cycle reentry [1]. An increasing number of ciliogenesis negative regulators have been identified, including multiple centriolar components that suppress cilia formation in cycling cells and proteins promoting cilia disassembly (reviewed by [1, 3]). However, the mechanisms underlying ciliogenesis are not yet fully uncovered.

Polyglutamylation is a conserved protein posttranslational modification (PTM) of ciliary axonemal microtubules (MTs) [4]. In this reversible procedure, tubulin tyrosine ligase-like (TTLL) polyglutamylases variably catalyze the attachment of a free glutamate to the γ-carboxyl of a glutamate residue in the protein primary sequence to form a branch point (initiation) or the subsequent addition of glutamate residues through α-carboxyl linkage to elongate the side chain (elongation) [5]. Conversely, the 6-member cytosolic carboxypeptidase (CCP) family catalyze the degradation and removal of the side chain. CCP5 is the only known enzyme that removes the branch point γ-carboxyl-linked glutamate after other CCP members shorten the side chain through degrading the α-carboxyl linkage [6, 7]. Although Nna1/CCP1, CCP4, and CCP6 similarly metabolize the α-carboxyl-linked glutamate in tubulin, they have distinct structural features [8, 9] and exhibit distinguishable enzyme kinetics for model peptide substrates as well as unequivalent biological functions [10].

The function of CCPs has been primarily linked to neuronal survival, as mutations of the prototypic Nna1/CCP1 underlie the loss of selective neurons in *Purkinje cell degeneration* (*pcd*) mutant mice [6, 11] and inherited early onset neurodegeneration in sheep [12] and humans [13, 14]. CCP5 and CCP6 mutant mice are susceptible to DNA virus infection due to the reduced activity of glutamylated cGAS [15]. *AGBL5*, the gene coding CCP5, is a retinitis pigmentosa causative gene [16, 17]. In mice, loss of this gene led to disrupted sperm flagella that are accompanied by abnormally organized microtubule arrays in the sperm manchette [7, 18]. Knocking out either CCP2 or CCP3 or even both, however, does not cause overt phenotypes, suggesting a possible functional redundancy among CCPs [19]. Although Nna1/CCP1 and CCP5 mutations cause ciliopathy-related anomalies, i.e., male infertility and photoreceptor degeneration in mouse or human [7, 16, 18, 20], the specific role of CCPs in cilia function remains largely unidentified.

The association between polyglutamylation and cilia function was established mainly through the role of TTLL polyglutamylases in ciliary architecture and motility. For instance, *Ttll6A* overexpression in *Tetrahymena* led to paralyzed cilia [21], while in a *Ttll9* mutant of *Chlamydomonas reinhardtii* (tpg1), the lack of a long polyglutamate side chain in α-tubulin impaired the interaction between MT and dynein and reduced flagellar motility [22]. In mouse, loss of *Ttll9* or *Ttll5* causes defects of different doublets in the axoneme 9 + 2 structure of sperm flagella, and therefore impaired motility [23, 24]. Ttll1 dysfunction led to the loss of beating asymmetry in tracheal epithelial cilia [25]. In contrast, fewer studies have examined the cilia-related function of CCPs. Among the four CCPs in zebrafish, only loss of CCP5 led to ciliary MT hyperglutamylation with associated motility defects that induce a spectrum of phenotypes characteristic of ciliopathy [26]. In *C. elegans*, the only 2 CCPs, ccpp1 and ccpp6, were both linked to ciliary sensor neuron function [27, 28]. Particularly, in mutants of ccpp1, the homolog of Nna1/CCP1, the architecture of the sensory cilia axoneme is disrupted [28]. Using ciliated cell models, CCP5 was found to be involved in regulating the length and transport of primary cilia [29]. However, it remains unknown whether CCPs play a role in ciliogenesis.

The CP110/CEP97 complex acts as a negative regulator of ciliogenesis, which caps the distal end of centrioles [30]. During ciliogenesis, the CP110/CEP97 complex at the mother centriole undergoes degradation through a proteasome pathway [31], allowing cilia assembly [30]. Depletion of CP110 or CEP97 induces cilia formation in cycling cells that can form cilia [30].

In this study, we show that CCP5 expression is transiently downregulated upon induction of ciliogenesis but gradually recovered after cilia formation. The transient downregulation of CCP5 expression is required for the initiation of ciliogenesis, which is independent of its enzyme activity. CCP5 interacts with CP110 and acts as a negative regulator of ciliogenesis by modulating CP110 expression. Similarly, CCP6 also modulates the protein level and location of CP110. Depletion of either CCP5 or CCP6 induces cilia formation in cycling cells, while co-depletion of CCP5 and CCP6 synergized this effect, suggesting their overlapped but distinct functions in suppressing cilia formation in cycling cells. After cilia formation, both CCP5 and CCP6 contribute to regulating cilia length, although they differently alter the length of polyglutamate side chains in the ciliary axoneme. This study demonstrates the dual role of two CCP members in cilia formation and length control, revealing a novel ciliogenesis regulatory mechanism mediated by CCPs, the demodifying enzymes of the conserved ciliary axoneme PTM — polyglutamylation.

## Results

### The dynamic expression of CCP5 during ciliogenesis

**Fig. 1:**
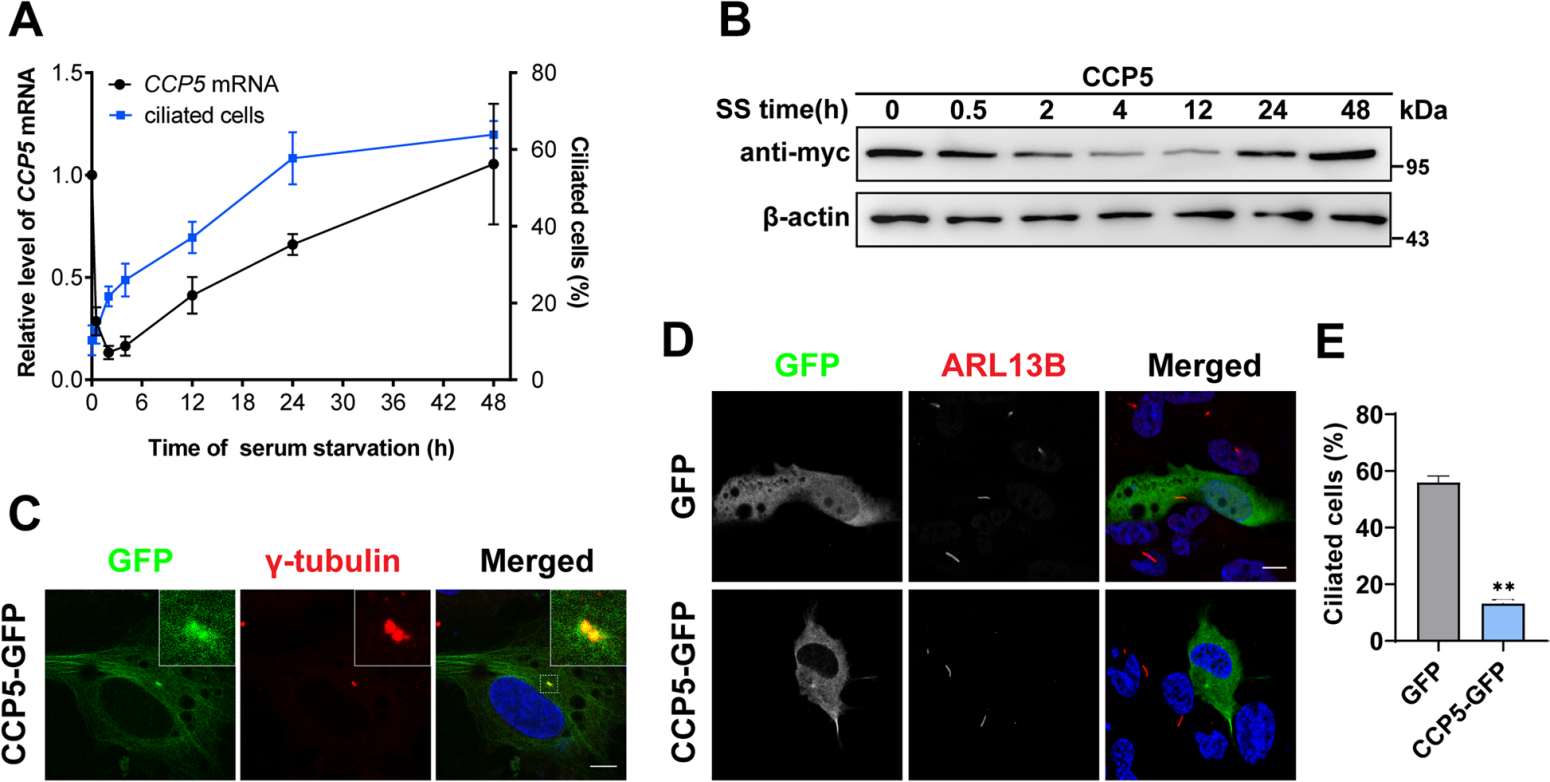
The correlation of CCP5 expression with ciliogenesis. **A** The time course of endogenous *CCP5* mRNA levels (black line) and the percentage of ciliated cells (blue line) in hTERT-RPE1 cells after serum starvation. The expression level of *CCP5* is dramatically reduced in 30 min after serum starvation and remains low after 4 h when ciliogenesis initiates, but gradually recovered to a level comparable to that before serum starvation at 48 h when cell ciliation is completed. The bars represent the mean ± s.d. from 3 independent experiments (Additional file 2). For the percentage of ciliated cells, at least 55 cells were analyzed per experimental condition (Additional file 2). **B** Representative immunoblot showed that in a HEK293T cell line stably expressing myc-CCP5, the exogenous CCP5 protein is reduced upon serum starvation and remains low 12 h later when cilia are rapidly formed (**A**). β-actin was used as a loading control. **C** Representative immunofluorescence images showed that in hTERT-RPE1 cells transfected with CCP5-GFP, CCP5 (green) is co-localized with and γ-tubulin (red). The nucleus is visualized by DAPI (blue) staining. The inserts are higher magnification views of the boxed regions. **D** After 24 h serum starvation, hTERT-RPE1 cells transfected with GFP or CCP5-GFP were stained with GFP (green), ARL13B (red), and the nucleus is visualized by DAPI (blue) staining. While the majority of GFP-transfected can form cilia, the CCP5-GFP-transfected cells rarely form cilia. **E** The quantification of the ciliated cells from 3 independent experiments with at least 30 cells analyzed per experimental condition in each experiment is shown in (**D**, Additional file 2). Error bars represent s.d. ∗  ∗ , *P* < 0.01, Student’s *t* test. Scale bars: 10 µm

Previous studies revealed the role of CCP5 in controlling the length of primary cilia [29]. However, its function in cilia formation was unknown. Using hTERT-RPE-1 (referred to RPE-1 below for simplicity) cells, which can form primary cilia upon serum starvation induction, we monitored the temporal expression pattern of CCP5 during ciliogenesis. The number of ciliated cells remains low in the first 4 h after serum starvation, but rapidly increases to ~ 40% after 12 h and reaches ~ 60% 24 h later (Fig. 1A, [32]). Interestingly, the level of CCP5 transcripts dramatically dropped more than 70% in the first 30 min after serum starvation, and remained low after 4 h. However, 12 h after serum starvation when the number of ciliated cells rapidly increases, the *CCP5* mRNA level was recovered. Between 12 and 48 h, the CCP5 mRNA level gradually reached a value comparable to that before serum starvation (Fig. 1A).

HEK293 cells also form cilia upon serum starvation induction. The temporal expression of *CCP5* in HEK293 cells correlates with ciliogenesis in a pattern resembling that in RPE-1 cells (Additional file 1: Fig. S1), suggesting a common phenomenon in ciliated cells. We wondered whether CCP5 expression can also be regulated on a protein level during ciliogenesis. Given the inefficiency of anti-CCP5 antibody to detect the low-level expression of endogenous CCP5, we sought to monitor changes in CCP5 protein level during ciliogenesis using a HEK293 cell line that stably expresses myc-tagged CCP5. Similar to the previous report, cells stably expressing CCP5 that passed selection can form cilia upon serum starvation at a rate comparable to that of control cells, though with reduced cilia length (Additional file 1: Fig. S1B-D, [29]). In these cells, the level of CCP5 protein was obviously reduced 4 h after serum starvation and remained at a low level at 12 h, when the number of ciliated cells rapidly increased. Twenty-four hours later, the level of CCP5 protein recovered to a level comparable to that before serum starvation (Fig. 1B). Taken together, these results suggest that during cilia formation, CCP5 expression is transiently downregulated on both transcriptional and translational levels.

### Overexpression of CCP5 negatively regulates cilia formation

The subcellular localization of CCP5 was analyzed in RPE-1 cells overexpressing CCP5-GFP. Despite its global expression in cytoplasm, the highly concentrated foci of CCP5-GFP signal were found to co-localize with that of γ-tubulin, a marker of centrioles (Fig. 1C), suggesting possible relevant function.

To determine whether transient downregulation of CCP5 expression is required for ciliogenesis, GFP-tagged CCP5 was transiently expressed in RPE-1 cells. In cells transfected with GFP, more than 50% of the cells formed cilia after serum starvation. However, the rate of ciliated cells is only about 10% in cells overexpressing CCP5-GFP (Fig. 1D, E). Therefore, transient overexpression of CCP5 inhibited cilia formation, suggesting that transient downregulation of CCP5 is necessary for ciliogenesis.

### The inhibition of CCP5 on cilia formation does not depend on its enzyme activity

**Fig. 2:**
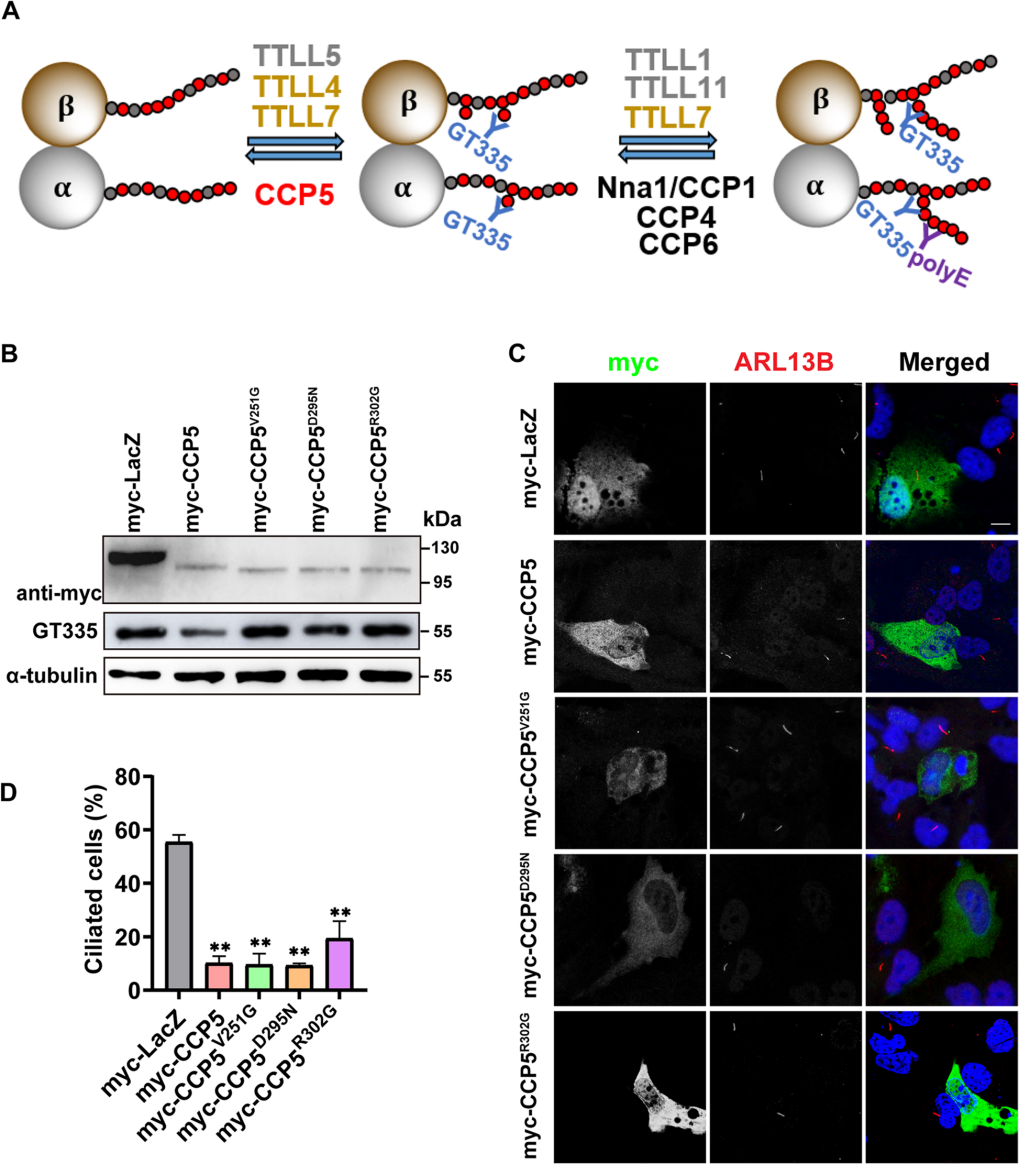
CCP5 suppresses ciliogenesis initiation independently of its enzyme activity. **A** A schematic representation shows the enzymes involved in tubulin polyglutamylation and that antibodies GT335 and polyE recognize the branch point glutamate and > 3 glutamate residues in chain respectively. **B** When porcine tubulin was incubated with the lysate of HEK293T cells transfected with myc-tagged LacZ, CCP5 or its mutants, CCP5 reduced the GT335 immunoreactivity compared with LacZ, indicative of its deglutamylation activity, while the CCP5 mutants did not alter the GT335 immunoreactivity, indicative of enzymatic inactivity. **C** After 24-h serum starvation, hTERT-RPE1 cells transfected with myc-tagged LacZ, CCP5, or CCP5 mutants CCP5^V251G^, CCP5^D295N^, or CCP5^R302G^ were immunostained with myc-tag (green) and ARL13B (red) and nuclei visualized with DAPI (blue). **D** Quantification of the ciliation in myc-positive cells (3 independent experiments; at least 30 cells analyzed per experimental condition, Additional file 2) revealed that similar to the wild-type CCP5, overexpression of the enzymatically inactive CCP5 mutants also suppressed cilia formation. Error bars represent s.d. ∗  ∗ , *P* < 0.01, Student’s *t* test. Scale bars: 10 µm

CCPs belong to the M14 metallocarboxypeptidase family, in which the enzyme activity is defined by 3 conserved motifs for Zn^2+^ binding, C-terminal carboxyl binding, and catalysis respectively [33]. To test whether the inhibitory role of CCP5 in ciliogenesis relies on its enzyme activity, CCP5 variants with mutated C-terminal carboxyl binding motif (CCP5^R302G^) or those identified in retinitis pigmentosa patients (CCP5^V251G^ and CCP5^D295N^) [16, 17] were generated. These variants were confirmed to be enzymatically inactive, reflected by their inability to reduce the immunosignal of GT335, an antibody recognizing the branch point glutamate, when using porcine tubulin as the substrate (Fig. 2A, B). When these mutants were transfected into RPE-1 cells individually, the number of ciliated cells was not significantly different from that transfected with wild-type CCP5 after serum starvation (Fig. 2C, D), suggesting a similar ciliogenesis inhibitory effect. Therefore, the suppression of CCP5 on ciliogenesis does not rely on its enzyme activity.

### Overexpression of CCP6, but not CCP1 or CCP4, also inhibits ciliogenesis

**Fig. 3:**
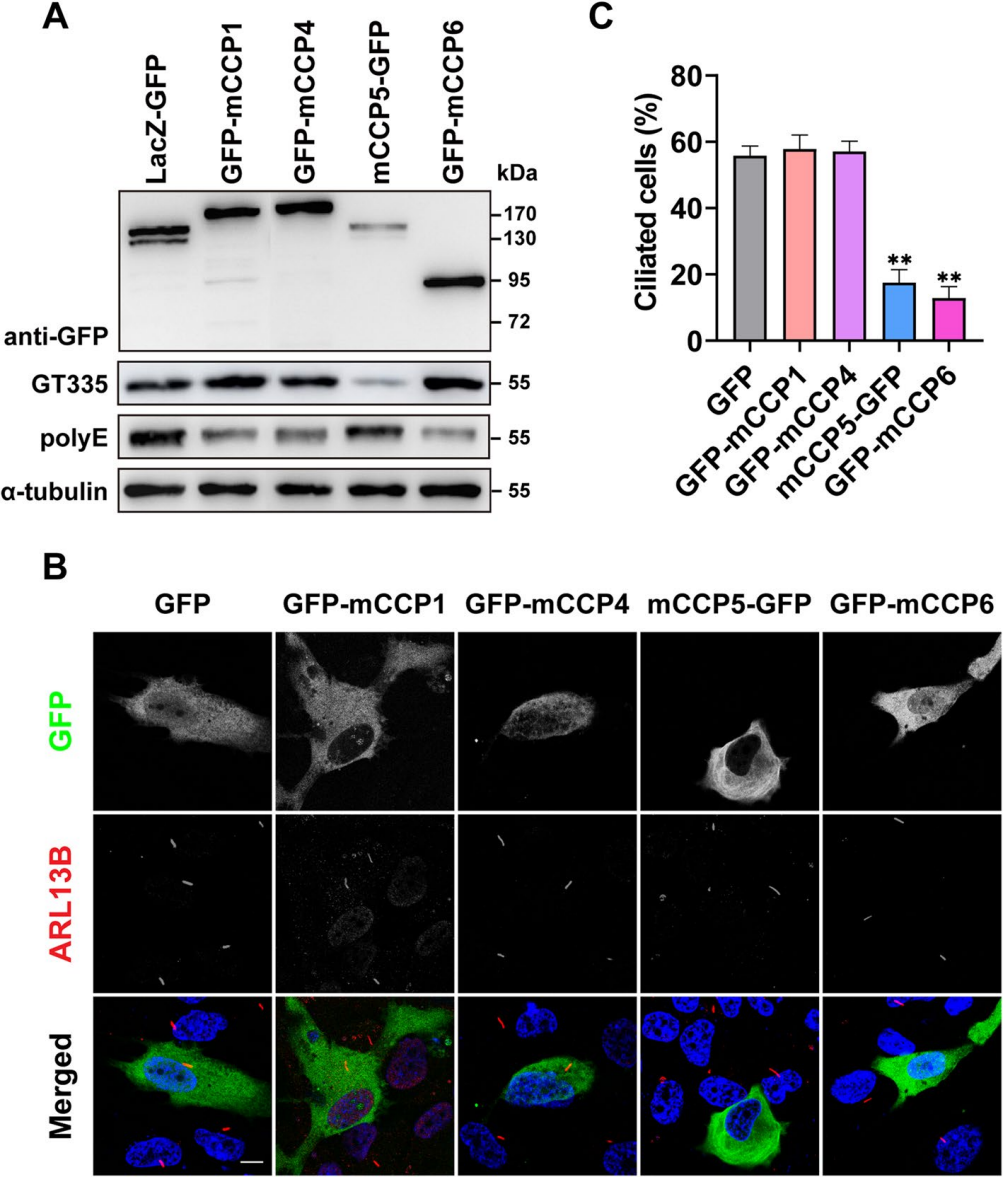
CCPs differentially regulate the initiation of cilia formation. **A** Immunoblotting analysis demonstrated the deglutamylation activity of GFP-tagged murine CCP1, CCP4, CCP5, and CCP6 when the lysates of their expressing HEK293T cells were incubated with porcine tubulin. CCP1, 4, and 6 reduced the immunoreactivity of polyE, which recognizes the long polyglutamate chain, while CCP5 specifically decreased the immunoreactivity of GT335, which recognizes the branch point glutamate. **B** hTERT-RPE1 cells transfected with GFP or GFP-CCPs were serum-starved for 24 h and immunostained with GFP (green) and ARL13B (red) and nuclei visualized with DAPI (blue). **C** Quantification of the ciliation in GFP-positive cells (3 independent experiments; at least 30 cells analyzed per experimental condition, Additional file 2) revealed that overexpression of CCP5 and CCP6 inhibited cilia formation. In contrary, overexpression of CCP1 and CCP4 did not alter the rate of ciliated cells. Error bars represent s.d. ∗  ∗ , *P* < 0.01, Student’s *t* test. Scale bars: 10 µm

The six CCPs are highly conserved among mammals. Transient overexpression of a mouse CCP5 in RPE-1 cells inhibited cilia formation to an extent comparable with its human orthologue (Figs. 1D, E, 3B, C). Nna1/CCP1, CCP4, and CCP6 can similarly degrade the α-carboxyl-linked glutamate in the polyglutamate side chain of tubulin, while CCP5 specifically metabolizes the branch point γ-carboxyl-linked monoglutamate [6, 7]. GFP-tagged Nna1/CCP1, CCP4, and CCP6 were confirmed to be active based on their ability to reduce the immunosignal of polyE that recognizes peptide chains with > 3 consecutive glutamate residues in tubulins (Fig. 3A). We wondered whether Nna1/CCP1, CCP4, and CCP6 can also affect cilia formation. Transient overexpression of CCP6 in RPE-1 cells reduced the number of ciliated cells to a level comparable to that of CCP5 after serum starvation. In contrast, neither Nna1/CCP1 nor CCP4 overexpression led to altered cell ciliation (Fig. 3 B, C). Therefore, CCPs are differentially involved in ciliogenesis, which is not related to their substrate selectivity.

### Similar to CCP5, CCP6 also negatively regulates the length of cilia in ciliated cells

**Fig. 4:**
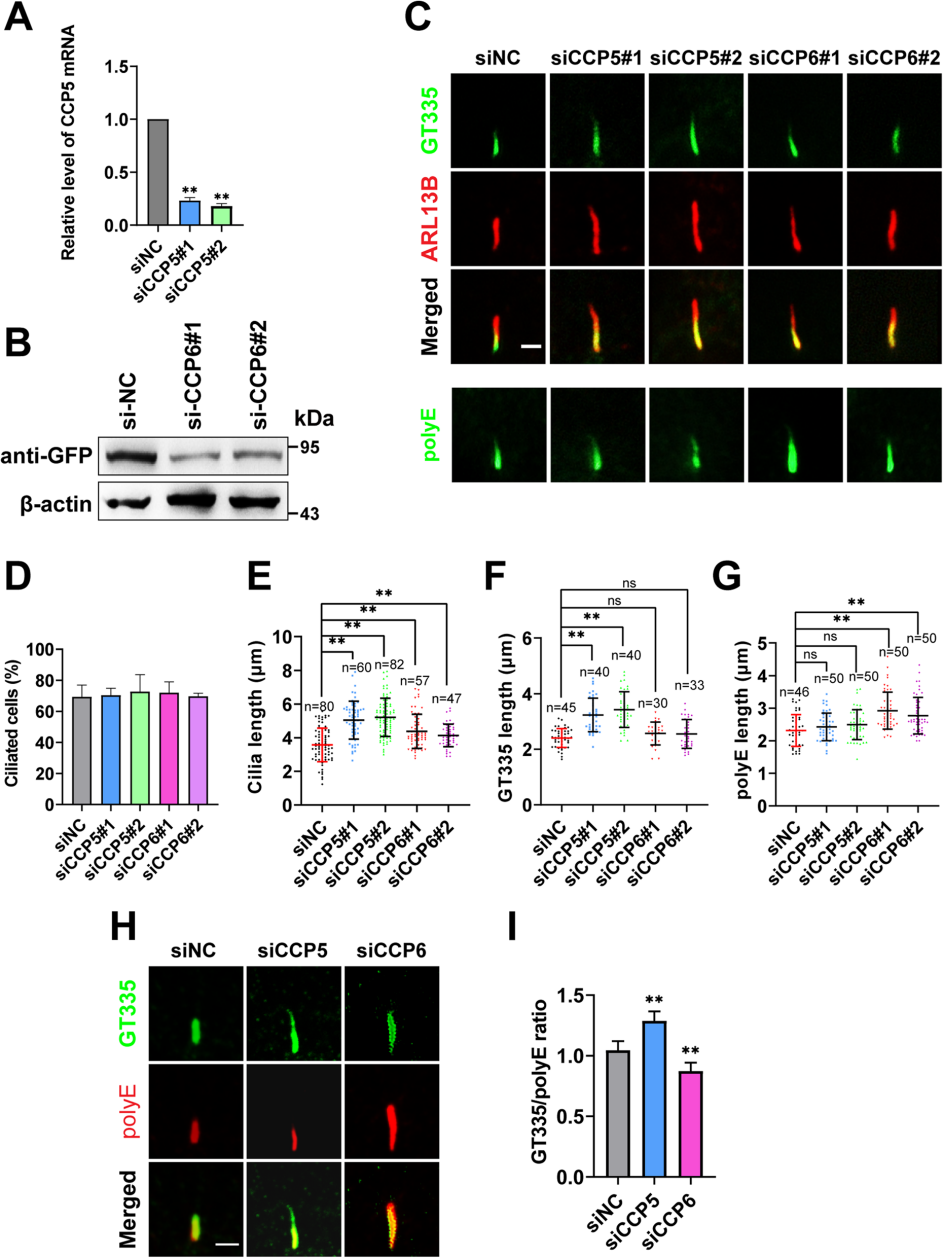
CCP5 and CCP6 differently regulate MT modifications in cilia. **A** qRT-PCR analysis confirmed that two independent siRNAs targeting human CCP5 (siCCP5#1, siCCP5#2) significantly reduced the CCP5 expression level in hTERT-RPE1 cells compared to cells transfected with non-targeting siRNA control (siNC). Data are means ± s.d. of 3 independent experiments (Additional file 2). **B** Immunoblotting analysis examined the efficiency of 2 CCP6 siRNAs (siCCP6#1, siCCP6#2) in depleting the overexpressed GFP-CCP6 in HEK293T cells. **C** Ciliary axoneme of hTERT-RPE1 cells treated with indicated siRNAs after 24-h serum starvation were detected with ARL13B (red) immunoreactivity for the length or GT335 or polyE (green) for the polyglutamylation levels. **D** Quantification of the ratio of ciliated cells showed that treatment with individual CCP5 or CCP6 siRNA did not affect cilia formation (3 independent experiments; at least 45 cells analyzed per experimental condition, Additional file 2). **E–G** Quantitative analysis of the length of cilia (**E**), axonemal GT335 (**F**), or polyE (**G**) in CCP5- or CCP6-depleted cells as exemplified in C. Each dot represents one cell. **H** Depletion of CCP5 or CCP6 differently increased axonemal glutamylation or polyglutamylation level as measured with GT335 (green) or polyE (red) immunoreactivity respectively. The length ratios between axonemal GT335 and polyE immunoreactivity were quantified in **I** (siNC: *n* = 45, siCCP5: *n* = 41, siCCP6: *n* = 31 cilia). Error bars represent s.d.. ∗  ∗ , *P* < 0.01, Student’s *t* test. Scale bars: 2 µm

A previous study showed that CCP5 depletion increased the length of cilia [29], while the role of CCP6 in cilia has not been reported, To determine whether CCP6 shares similar function with CCP5 in cilia, siRNAs targeting distinct coding regions of CCP5 or CCP6 were designed. The efficiencies of CCP5 siRNAs were validated for their ability to deplete endogenous gene expression (Fig. 4A), However, using qRT-PCR, *CCP6* mRNAs were not convincingly detectable in either RPE-1 or HEK293 cells, consistent with previous findings that *CCP6* transcripts remain at a low level hardly detectable in multiple cell lines [34]. Therefore, the efficiencies of CCP6 siRNAs were confirmed based on their ability to reduce the level of overexpressed proteins (Fig. 4B).

In line with the previous study [29], the ciliation of RPE-1 cells was not affected by CCP5 siRNA treatment, but the length of cilia was significantly increased after serum starvation (Fig. 4C–E). Similarly, knocking down CCP6 did not affect the ratio of ciliated cells either, but also significantly increased the length of cilia, although to a lesser extent than silencing CCP5 (Fig. 4C–E). It is possible that the effects of siCCP6 on cilia length resulted from its mistargeting to other CCPs. To this end, we assessed the expression of CCP5 and CCP1, another CCP member with detectable expression level [34], in CCP6 depleted cells using qRT-PCR. As shown in Fig. S2A, the expression of neither CCP1 nor CCP5 was affected by CCP6 depletion. Consistently, siCCP6 did not alter the protein level of CCP5 in cells stably expressing myc-CCP5, and vice versa (Additional file 1: Fig. S2B), excluding the possibility of off-targeting to CCP5 or CCP6 by siCCP6 and siCCP5 respectively. Therefore, similar to CCP5, CCP6 not only negatively regulates ciliogenesis, but also contributes to maintaining the length of cilia.

### CCP5 and CCP6 differently regulate MT modifications in cilia

CCP6 shortens the long polyglutamate side chain, while CCP5 specifically catalyzes the removal of the branch point monoglutamate. We first assessed whether depletion of CCP5 or CCP6 can lead to an increase in the overall glutamylation level of the cells. However, the signals of GT335 were hardly detectable in RPE-1 cell lysate by Western blotting, even after CCP5 or CCP6 were depleted, which is supposed to increase the glutamylation level (Additional file 1: Fig. S2C). These results suggested that RPE-1 cells have a low basal tubulin glutamylation level, similar to HEK and Hela cells.

We then use immunofluorescence to assess whether depletion of CCP5 or CCP6 altered MT polyglutamylation in cilium with GT335 and polyE antibodies, which recognize the branch point glutamate and the long-chain polyglutamate respectively [6, 35]. Depleting either CCP5 or CCP6 increased the length of cilia after serum starvation, based on the signal of ARL13B (Fig. 4C, E), a protein localized on the ciliary membrane [36]. However, compared with the control, knocking down CCP5 only significantly increased the length of GT335 signal in the cilia, but not that of polyE (Fig. 4C, E, F). In contrast, ablation of CCP6 only significantly increased the length of polyE signal but not that of GT335 (Fig. 4C, F, G). As a consequence, the ratio of GT335 signal length to that of polyE increased in CCP5-depleted cilia, but decreased in CCP6-depleted cilia (Fig. 4H,I). These results indicate that (1) CCP5 and CCP6 are both involved in the PTM of axonemal MTs; and (2) the increased monoglutamylated population upon CCP5 depletion did not further increase the long side-chain MT, suggesting a tightly controlled length of polyglutamate side chain in ciliary axonemal MTs.

### CCP5 interacts with ciliogenesis negative regulator CP110

To understand how CCP5 and CCP6 fulfill their function in regulating cilia formation, we set out to determine their interaction partners using cells stably expressing CCP6, as CCP6 is almost solely comprised of the conserved N-domain and CP domain of the CCP family [8] and presumptively represents the common functional region for CCP5 and CCP6. Similar to cells stably overexpressing CCP5, CCP6 overexpressing stable cell lines that passed selection also exhibited shortened cilia without altered ciliation ratio (Fig. S1B, C, D). Co-immunoprecipitation-mass spectrometry analysis identified CP110 as one of the top hits for potential interactors based on the Sum PEP score ranking (Additional file 3: Table S2).

**Fig. 5:**
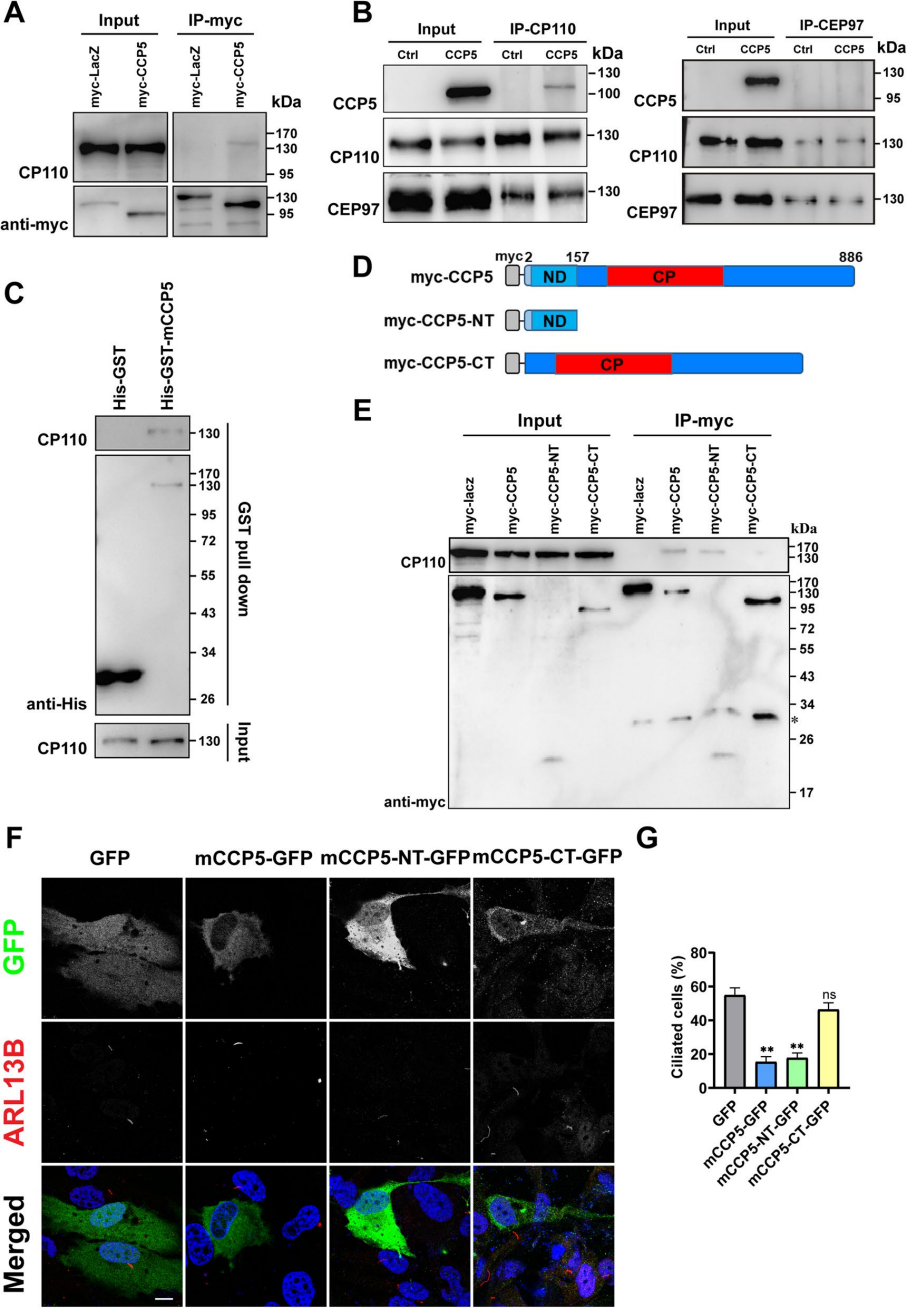
CCP5 interacts with CP110 through its N-terminus. **A** The lysates of HEK293T cells stably expressing myc-tagged LacZ or CCP5 were immunoprecipitated with anti-myc affinity beads. Protein levels of endogenous CP110 and myc-CCP5 in immunoprecipitants and cell lysates were detected. CP110 can be immunoprecipitated from cells expressing myc-CCP5 but not myc-LacZ. **B** Lysates of control or HEK293T cells stalely expressing CCP5 were immunoprecipitated with CP110 or CEP97 polyclonal antibodies. CCP5 can be co-immunoprecipitated with CP110, but not CEP97. As a control, CP110 was co-immunoprecipitated with CEP97. **C** Direct interaction between CCP5 and endogenous CP110 detected by GST pull-down assay. HEK293T cell lysates were incubated with glutathione resin bound with His-GST or His-GST-CCP5 proteins. The eluent obtained by GSH competition was examined using CP110 antibody. **D** Schematic representation of full-length CCP5 and the indicated CCP5 truncations. ND, N-domain; CP: carboxypeptidase domain. **E** The myc-LacZ or full-length or truncated CCP5 was expressed in HEK293T cells and immunoprecipitated with anti-myc affinity beads. The interaction with endogenous CP110 was determined by indicated antibodies. An unspecific band from IP appears at the position marked with *. **F** hTERT-RPE1 cells transfected with GFP, CCP5-GFP, or CCP5 truncations with GFP tag were serum-starved for 24 h and immunostained with GFP (green) and ARL13B (red) and nuclei were visualized with DAPI. Representative images showed that similar to CCP5 wild-type, its N-terminal, but not C-terminal truncation is sufficient to inhibit cilia formation. **G** Quantification of the ciliation in GFP-positive cells shown in F (3 independent experiments; at least 30 cells analyzed per experimental condition, Additional file 2)

CP110 is a centriolar protein which caps the distal end of centrioles in complex with CEP97 and acts as a negative regulator of ciliogenesis [30]. CP110 overexpression inhibits ciliogenesis [30], similar to the effects of overexpression of CCP5 or CCP6. Further validation experiments revealed that endogenous CP110 can be successfully co-immunoprecipitated by myc-CCP6, but CCP6 was not co-immunoprecipitated by CP110 antibody (data not shown), suggesting a possibly indirect interaction between CCP6 and CP110. The notion that CCP5 interacts with CP110 is supported by the observation that endogenous CP110 can be co-immunoprecipitated from the lysate of cells stably expressing myc-CCP5, but not from that expressing myc-LacZ (Fig. 5A). Conversely, CCP5 can also be co-immunoprecipitated with CP110, but not with the CP110 interacting protein — CEP97 (Fig. 5B).

The interaction between CCP5 and CP110 was further validated using an orthogonal method. HEK293 cell lysate was passed through a column immobilized with purified GST-mCCP5 that was expressed in Sf9 insect cells. Indeed, CP110 can be co-eluted with GST-mCCP5, but not with GST (Fig. 5C), further supporting the proposed interaction between CCP5 and CP110. In addition, co-immunostaining of GFP and CP110 in CCP5-GFP expressing cell demonstrated a partial colocalization between the two proteins. While CP110 appears as two little dots at centrioles, CCP5-GFP foci are larger in size (Additional file 1: Fig. S3).

### CCP5 interacts with CP110 through its N-terminus

We further determined the specific region of CCP5 that binds to CP110. The CCP family contains a unique “N-domain” of unknown function that resides N-terminal to the carboxypeptidase (CP) domain [8]. The myc-tagged versions of the N- and C- terminal domains of CCP5, which contain the N- and CP domains respectively (Fig. 5D) were generated. We found that the N-terminal domain was able to co-immunoprecipitate CP110, but not the C-terminal domain (Fig. 5E). These observations indicate that CCP5 interacts with CP110 through its N-terminus.

### The N-terminus of CCP5 is sufficient to inhibit cilia formation

We wondered whether the ability of CCP5 to inhibit ciliogenesis relies on its N-terminus. Plasmids encoding GFP-tagged CCP5 N-terminal domain (CCP5-NT) or C-terminal domain (CCP5-CT) (Fig. 5D) were transfected into RPE-1 cells. The number of ciliated cells in CCP5-NT expressing cells were significantly reduced to a level comparable with that expressing full-length CCP5 after serum starvation, while the rate of ciliated population in CCP5-CT expressing cells is similar to that of the control (Fig. 5F,G). Therefore, CCP5 fulfills its function to suppress cilia formation through its N-terminus instead of its C-terminus, consistent with our observation that CCP5 suppresses cilia formation independently of its enzyme activity (Fig. 2).

### CCP5 and CCP6 regulate CP110 level

**Fig. 6:**
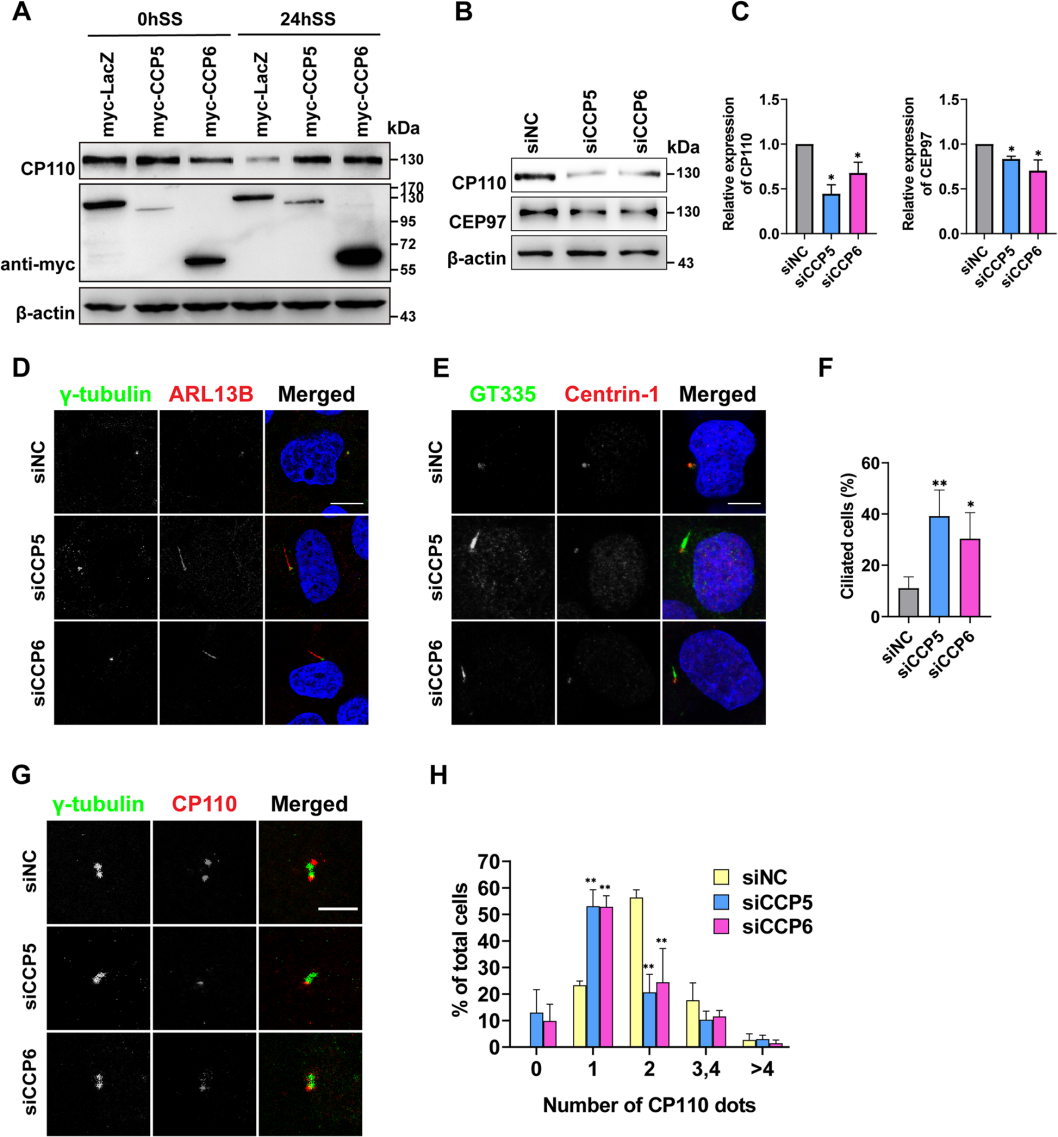
CCP5 and CCP6 are required to localize CP110 at mother centrioles in cycling cells. **A** Endogenous CP110 expression levels determined by immunoblotting in control (LacZ), CCP5, or CCP6 overexpressed HEK293T cells before and after serum starvation. In LacZ overexpressed cells, CP110 expression level was reduced after 24 h serum starvation, while in cells overexpressing CCP5 or CCP6, CP110 levels remain comparable before and after serum starvation. β-actin was used as a loading control. **B** In HEK293 cells treated with CCP5 or CCP6 siRNAs, the endogenous CP110 and CEP97 expression was reduced compared with that in control siRNA (siNC) treated cells. **C** Quantification of CP110 or CEP97 protein levels from results exemplified in B (3 independent experiments, Additional file 2). The intensities of the immunoblotting bands were normalized to those of β-actin. **D** hTERT-RPE1 cells transfected with control (siNC), CCP5, or CCP6 siRNA in the presence of serum were immunostained with γ-tubulin (green), ARL13B (red), and nuclei visualized with DAPI (blue). Depletion of CCP5 or CCP6 induced ciliation in cycling cells. **E** The elongated structure formed in CCP5 or CCP6 depleted hTERT-RPE1 cells in the presence of serum was positive for another cilia marker GT335 (green), but devoid of the centriole protein Centrin-1 (red). Nuclei were visualized with DAPI (blue). **F** Quantification of the ciliated cells in CCP5 or CCP6 depleted hTERT-RPE1 cells in the presence of serum as exemplified in D. Data were obtained from 3 independent experiments with at least 30 cells analyzed in each experiment per experimental condition (Additional file 2). **G** hTERT-RPE1 cells transfected with control (siNC), CCP5, or CCP6 siRNA in the presence of serum were immunostained with γ-tubulin (green), CP110 (red), and nuclei visualized with DAPI (blue). In control group, cells with 2 dots of CP110 were commonly seen, but in CCP5- or CCP6-depleted cells, those with only 1 dot of CP110 became more common, where γ-tubulin is still present as 2 dots, indicative of the integrity of centrioles. **H** Quantification of the percentage of cells with the indicated number of CP110 dots in hTERT-RPE1 cells transfected with control (siNC), CCP5, or CCP6 siRNA in the presence of serum (3 independent experiments; at least 25 cells analyzed per experimental condition in each experiment, Additional file 2). Error bars represent s.d., ∗ , *P* < 0.05; ∗  ∗ , *P* < 0.01 Student’s *t* test. Scale bars: 10 µm (**D, E**); 4 µm (**H**)

During ciliogenesis, CP110 in complex with CEP97 at the mother centrioles undergoes degradation, allowing the initiation of cilia assembly [31]. Indeed, CP110 protein level in cells transiently overexpressing myc-lacZ was greatly reduced 24 h after serum starvation (Fig. 6A). However, in cells overexpressing CCP5 or CCP6, CP110 protein levels remained comparable before and after serum starvation (Fig. 6A), in line with their inability to form cilia. Conversely, in CCP5- or CCP6-depleted cells, the level of CP110 was significantly reduced, while the level of CEP97 was also reduced, although to a less extent (Fig. 6B,C). Taken together, these observations suggest that the expression of CCP5 and CCP6 regulates CP110 level.

### Depletion of CCP5 or CCP6 induces cilia formation in cycling RPE-1 cells

Upon CP110 depletion, premature cilia can be formed in cycling cells able to form cilia [30, 32, 37], while loss of proteins that stabilize the CP110-CEP97 complex, such as Kif24, and MPP9 resulted in similar abnormalities [32, 37]. As depletion of CCP5 or CCP6 reduced the level of CP110, we wondered whether knocking down CCP5 or CCP6 is able to induce cilia formation in cycling RPE-1 cells. Without serum starvation, normally ~ 10% cells can grow cilia in RPE-1 cells. However, depletion of CCP5 or CCP6 significantly increased the number of ciliated cells to ~ 30% respectively (Fig. 6D–F). These hair-like structures better resemble cilia rather than elongated centrioles as they are immunopositive for glutamylated MT (GT335), but are devoid of centrin-1, a centrosome marker (Fig. 6E). In CCP5- or CCP6-depleted cells, overexpression of the corresponding mouse homologs not only led to the recovery of CP110 protein levels (Additional file 1: Fig. S4A, B), but also reduced the rate of ciliated cells in cycling cells to a normal level (Additional file 1: Fig. S4C, D), confirming the observation of abnormal ciliation upon CCP5 or CCP6 silencing. Therefore, ablation of CCP5 or CCP6 induces cilia formation in cycling RPE-1 cells.

### CCP5 and CCP6 are required to localize CP110 at mother centrioles in cycling cells able to form cilia

We wondered whether the localization of CP110 in growing RPE-1 cells was affected by ablation of CCP5 or CCP6. In general, CP110 exists as two or four dots in cycling cells, while during ciliogenesis, CP110 is detached from the mother centriole, and the number of CP110-positive dots in cells is often one or three [30]. Interestingly, in CCP5- or CCP6-depleted cycling RPE-1 cells, the population that displayed one CP110 immunopositive dot was significantly increased at the expense of cells with two CP110 dots when compared to the control (Fig. 6G,H). The persistence of γ-tubulin dots in CCP5- or CCP6-depleted cells indicated the integrity of centrioles (Fig. 6G). Taken together, these observations suggest that CCP5 and CCP6 are required to localize CP110 at mother centrioles in cycling cells.

### CCP5 and CCP6 synergistically regulate cilia formation

**Fig. 7:**
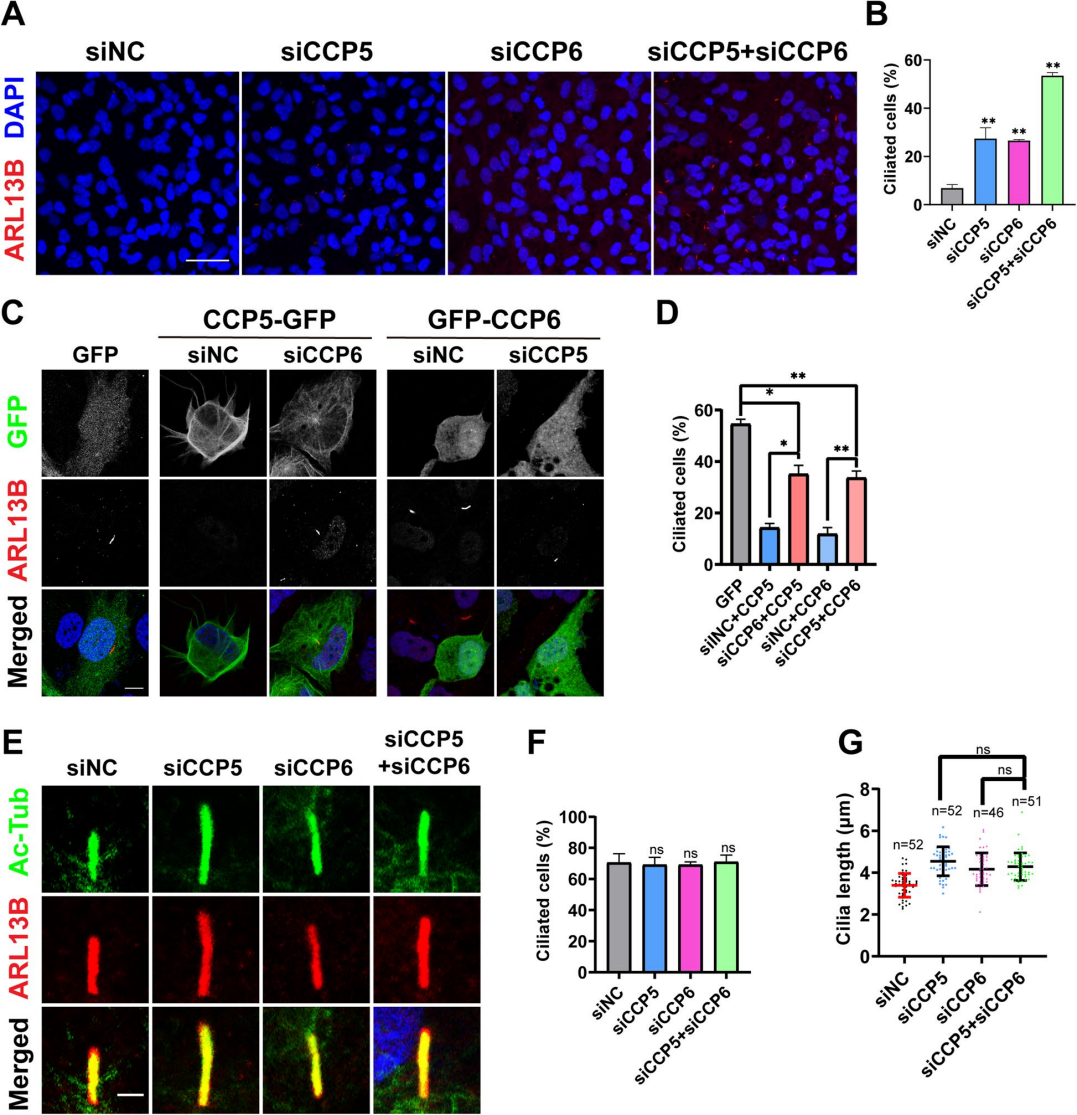
CCP5 and CCP6 synergistically inhibit cilia formation, but not the length of cilia. **A** Co-depletion of CCP5 and CCP6 in cycling hTERT-RPE1 cells synergized the abnormal cilia formation. hTERT-RPE1 cells transfected with control (siNC), CCP5, or CCP6 siRNA or both in the presence of serum were immunostained with ARL13B (red) to detect cilia and nuclei were visualized with DAPI (blue). **B** Quantification of the ciliation in siRNA-treated cells exemplified in A (3 independent experiments; at least 65 cells analyzed per experimental condition, Additional file 2). **C** Depletion of CCP6 or CCP5 partially rescued the inhibited cilia formation caused by respective CCP5 and CCP6 overexpression in quiescent hTERT-RPE1 cells. Cells transfected with GFP, GFP-tagged CCP5 or CCP6 and indicated siRNAs were serum-starved for 24 h and then immunostained with GFP (green), ARL13B (red), and nuclei were visualized with DAPI (blue). **D** Quantification of the ciliation in GFP-positive cells exemplified in C (3 independent experiments; at least 30 cells analyzed per experimental condition, Additional file 2). **E** Ciliary axoneme of quiescent hTERT-RPE1 cells transfected with control (siNC), CCP5 (siCCP5), or CCP6 (siCCP6) siRNA or both siRNAs were visualized by immunoreactivity for acetylated-tubulin (Ac-Tub, green) or ARL13B (red). Depletion of either CCP5 or CCP6 increased the ciliary length compared to the control, but co-depletion of CCP5 and CCP6 did not lead to further increase in cilia length. **F** Quantification of the ciliated cells recognized by ARL13B immunoreactivity in conditions shown in E (3 independent experiments; at least 45 cells analyzed per experimental condition, Additional file 2). **G** Quantitative analysis of the cilia length detected by ARL13B immunoreactivity as exemplified in E (Additional file 2). There is no significant difference between the CCP5 and CCP6 co-depleted group with that depleted either alone. Each dot represents one cell. Scale bars: 20 µm (**A**); 10 µm (**C**); 2 µm (**E**); 5 µm (**H**). Error bars represent s.d.. ∗ , *P* < 0.05; ∗  ∗ , *P* < 0.01; ns, no significant difference, Student’s *t* test

As CCP5 and CCP6 similarly affect the localization of CP110 at mother centrioles, we wondered whether they share a common pathway to inhibit cilia formation in cycling cells. Without serum starvation, depleting either CCP5 or CCP6 in RPE-1 cells can increase the number of ciliated cells from ~ 10 to ~ 30%. Strikingly, depleting both CCPs in cycling cells caused the number of ciliated cells to reach about 50%, a rate similar to that obtained with serum starvation induction (Fig. 7A,B). These observations suggest that CCP5 and CCP6 synergize the suppression of cilia formation in cycling RPE-1 cells. Moreover, in CCP5 and CCP6 co-depleting cells, the level of CP110 was not further reduced compared with those with either CCP singly depleted (Additional file 1: Fig. S5). Therefore, although CCP5 and CCP6 can similarly attenuate CP110 levels and inhibit cilia formation in cycling cells, they suppress the formation of cilia in cycling cells through overlapped but distinctive pathways.

To further investigate the functional correlation between CCP5 and CCP6, we tested whether depleting one can rescue the inhibition of cilia formation by another. When cells were transfected with CCP5 and a control siRNA (siNC), the percentage of ciliation is only ~ 10% after serum starvation, much lower than the control (~ 60%). In CCP6-depleted cells, however, overexpression of CCP5 increased the percentage of ciliated cells to ~ 35% (Fig. 7C, D). Conversely, knocking down CCP5 also partially rescued the inhibition of cilia formation by overexpression of CCP6 (Fig. 7C, D). These results further support that CCP5 and CCP6 have partially overlapped function in regulating cilia formation.

### CCP5 and CCP6 regulate the length of cilia through a common pathway

 Since depleting either CCP5 or CCP6 increased the length of cilia in RPE-1 cells after serum starvation, we wondered whether they also have a synergistic effect on length control in cilia. However, when CCP5 and CCP6 were ablated simultaneously, the length of cilia was not further increased compared to the depletion of either one individually. Likewise, the number of ciliated cells after serum starvation was also similar for the singly and doubly ablated samples (Fig. 7E–G), unlike the case in cycling cells (Fig. 7A). These observations suggest that (1) CCP5 and CCP6 probably use a common pathway to control the length of cilia; (2) the increased cilia length in quiescent cells due to depletion of CCP5 or CCP6 was not attributable to the premature formation of cilia before serum starvation, as otherwise increased cilia length would be expected when both CCP5 and CCP6 were depleted. Thus, CCP5 and CCP6 partially rely on one another to suppress cilia formation, but apparently use a common pathway to control the length of cilia.

### The dual role of CCP5 and CCP6 in ciliogenesis and cilia length control

**Fig. 8:**
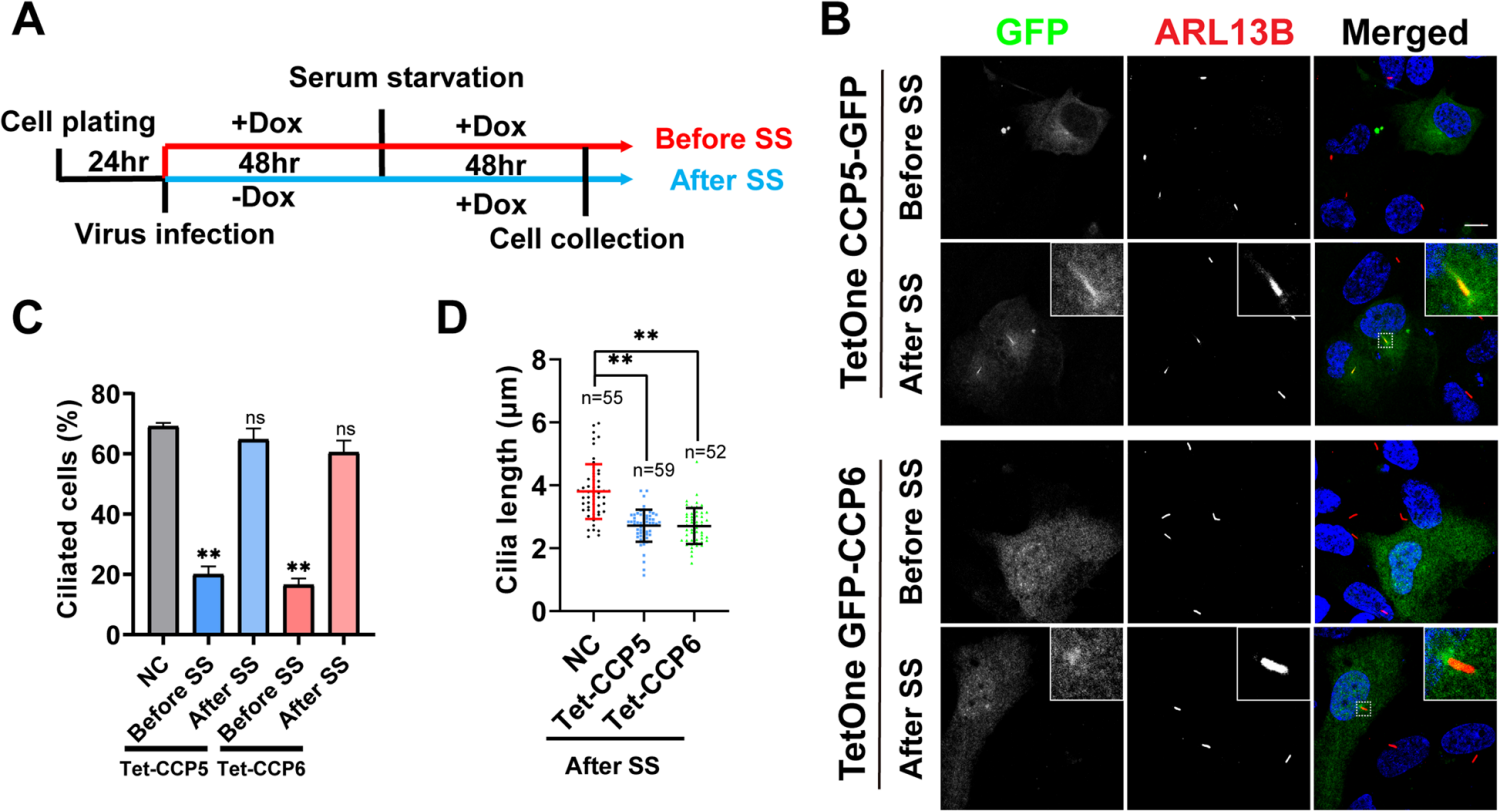
Segregation of the dual function of CCP5 and CCP6 in ciliogenesis suppression and ciliary length control using Tet-On inducible expression system. **A** Experimental scheme for inducible overexpression of CCP5 or CCP6 before or after ciliogenesis. hTERT-RPE1 cells were cultured for 24 h and infected with Tet-on inducible CCP5 or CCP6 expression virus for 48 h. The cells were then changed to serum-free medium and continuously cultured for another 48 h. To induce CCP expression before ciliogenesis, doxycycline (Dox) was added at the beginning of infection (Before SS, red) and remained after serum starvation, while to induce CCP expression after ciliogenesis doxycycline was added upon serum starvation (After SS, blue). **B** hTERT-RPE1 cells were infected with virus-containing Tet-On controlled CCP5-GFP or GFP-CCP6 expressing plasmid and the expression was induced at the time points shown in **A**. Cells were immunostained with GFP (green) and ARL13B (red), and nuclei were visualized with DAPI (blue). **C** Quantification of the ciliation in GFP-positive cells representatively shown in **B** with the untreated hTERT-RPE1 cells used as the control. When CCP5 or CCP6 expression is induced before serum starvation, the ratios of ciliated cells were significantly reduced. When their expression was induced after serum starvation, the ratios of ciliated cells were not affected (3 independent experiments; at least 30 cells analyzed per experimental condition, Additional file 2). **D** Quantitative analysis of the cilia length of GFP-positive cells when CCP5 or CCP6 expression were induced after serum starvation (after SS) as shown in B. Each dot represents one cell (Additional file 2). Error bars represent s.d.. ∗  ∗ , *P* < 0.01. Student’s *t* test. Scale bars: 10 µm

Given the above-determined role of CCP5 and CCP6 in ciliogenesis and cilia length control, we proposed that these CCPs execute segregated functions before and after cilia formation. In order to further verify this notion, a Tet-on system was applied to induce CCP5 or CCP6 overexpression at two separate stages of cilia development. The tightly controlled overexpression of CCP5 and CCP6 upon doxycycline (Dox) induction was validated with Western blotting (Additional file 1: Fig. S6). After infection with lentivirus expressing CCP5 or CCP6, their expression was induced before or after serum starvation (Fig. 8A). Indeed, induction of CCP5 or CCP6 overexpression before serum starvation effectively inhibited ciliation (Fig. 8B, C), consistent with the results from transient overexpression of each protein (Figs. 1D, 3 and 7). In contrast, induction of CCP5 or CCP6 overexpression after serum starvation did not affect the ratio of ciliated cells, but led to a reduced length of cilia (Fig. 8B-D). These results confirmed the dual role of CCP5 and CCP6 — they negatively regulate ciliogenesis, but also contribute to control the length of cilia after cilia formation. Notably, when induced after ciliogenesis, both CCP5 and CCP6 tend to gather around the basal body. CCP5 is also able to enter the cilia (Fig. 8B), consistent with the previous report [29]. However, the overexpressed CCP6 could form concentrated foci at the basal body but was not detected in cilia (Fig. 8B). Nevertheless, these results underscore the dual role of CCP5 and CCP6 during different stages of cilia development. They suppress cilia formation in cycling cells and regulate the length of cilia after cilia are formed.

## Discussion

Polyglutamylation is a conserved reversible PTM in the ciliary axoneme. The role of its writers has been linked to proper ciliary architecture formation and motility, while the cilia-related function of polyglutamylation erasers, CCPs, remains largely elusive. Here, we report that two CCP family members, CCP5 and CCP6, are novel negative regulators of ciliogenesis and also play a role in limiting cilia length after cilia formation. Particularly, CCP5 and CCP6 retain the level of the centriole-capping protein CP110. Depletion of either induces cilia formation in cycling cells, resembling the effects of depleting CP110. CCP5 interacts with CP110 through its N-terminal region, which alone is sufficient to inhibit ciliogenesis. CCP5 and CCP6 suppress formation of cilia in cycling cells through partially overlapping mechanisms. It is likely that they control cilia length through a common pathway, although differentially regulating the length of polyglutamate chain in the ciliary axoneme.

The cilia-related function of CCPs has been predicted based on phylogenetic studies and their localization at cilia-related structures [38]. However, it was unknown whether CCPs are involved in ciliogenesis. In this study, we show that a transient downregulation of CCP5 expression is required for ciliogenesis. Its temporal expression pattern correlates with ciliogenesis, which reflects its segregated functions in suppressing cilia formation in cycling cells and regulating the axonemal PTM after cilia formation. CCP5 fulfills the former function through its N-terminal domain and independently of its enzyme activity, while it achieves the latter role through its catalytic activity to modify the axoneme MTs. Polyglutamylation tends to increase the stability of MT [39], which probably accounts for the role of CCP5 and CCP6 in regulating the length of cilia. In line with our findings, a previous study used a specialized ciliary-targeting tool to recruit the CCP5 catalytic domain into cilia and found that increased deglutamylation activity in cilia reduced ciliary length, without altering cell ciliation [40]. Interestingly, two retinitis pigmentosa causative mutations located in the carboxypeptidase domain of CCP5 (CCP5^V251G^ [16] and CCP5^D295N^ [17]) did not affect its role in suppressing cilia formation, suggesting a pathological mechanism related to CCP5 activity.

Multiple proteins have been identified as ciliogenesis negative regulators through interaction with the CP110-CEP97 complex. For instance, Kif24, a kinesin 13 family member, not only stabilizes CP110 to prevent cilia assembly at inappropriate times, but also counteracts microtubule polymerization by remodeling microtubules at the distal end of centrioles that could otherwise lead to premature formation of cilia [37]. In addition, MPP9 recruits the CP110-CEP97 complex to the mother centriole through direct binding of CEP97 [32]. The present study extended the list of such proteins by uncovering that two CCP members can modulate CP110 levels and negatively regulate ciliogenesis. Notably, different from the above-mentioned ciliogenesis negative regulators, whose expression levels remain low after cilia formation [31, 32], CCP5 and CCP6 expression levels are elevated when ciliation is completed (Fig. 6A), pointing to the unique role of CCPs in cilia development. Our findings suggested a likely scenario in which CCP5 remains at a high level during cell cycling to suppress cilia formation, while its expression is wiped away through a yet unidentified mechanism to permit cilia formation during ciliogenesis, but then restored after cilia are formed. A genome-wide RNAi screening indicated that mRNA processing and the ubiquitin–proteasome system (UPS) are commonly involved in ciliogenesis [41]. Whether such machineries contribute to the removal of CCPs during ciliogenesis requires further investigation.

Depletion of either CCP5 or CCP6 led to a reduction of CP110 protein and about a three-fold increase in the number of ciliated cells in cycling RPE-1 cells, a ratio similar to that seen after depleting CP110 [30]. Strikingly, co-depletion of CCP5 and CCP6 increased the number of ciliated cells by about five-fold, while the level of CP110 was not further reduced compared to that with single enzyme depletion, suggesting possible involvement of additional ciliogenesis regulatory mechanisms. Notably, despite its obvious role in suppressing cilia formation in cycling cells, the mRNA level of CCP6 remains low in RPE-1 and HEK293 cells, possibly reflecting a rather transient and/or spatially restricted expression pattern. Indeed, CCP6 expression was found to be restricted to the centrioles of Hela cells at interphase [38]. Therefore, the synergistic suppression of cilia formation in cycling cells by CCP5 and CCP6 might also be attributed to their functions at distinct cell cycle phases.

Based on sequence similarity, 6 CCP members were subcategorized into 3 clades, with CCP5 and CCP6 in one and Nna1/CCP1 and CCP4 in another [27]. Like CCP6, Nna1/CCP1 and CCP4 also catalyze the degradation of α-carboxyl-linked glutamate in the polyglutamate side chain. However, overexpression of neither inhibited cilia formation in RPE-1 cells. The CCP family possesses a unique conserved N-domain of undefined function that resides N-terminal to their CP domain. We showed that the N-domain of CCP5 mediates its binding to CP110, exemplifying the regulatory role of CCP N-domain through interaction with other proteins. It remains unknown how the N-domain of CCP5 contributes to modulate the level of CP110. Our preliminary data show that depleting CP110 does not affect the protein level of CCP5 in its stable cell line (data not shown), suggesting that CP110 is possibly downstream of CCP5. Depletion of CCP5 or CCP6 can reduce the mRNA level of CP110 in HEK293 cells (data not shown), suggesting that CCP5 and CCP6 may also regulate a transcription factor of CP110. However, the location-specific loss of CP110 at the centriole upon CCP5 or CCP6 depletion is more likely related to a regulatory mechanism in the protein level. One possibility for CCP5 to conduct this role is that when ciliogenesis initiates, the N-terminus of CCP5 is subjected to some modifications that promote its own degradation and/or release CP110 from binding for further location-specific removal. Although CP110 was initially immunoprecipitated as a possible interactor of CCP6, the unsuccessful reverse IP of CCP6 with CP110 antibody discouraged us from further pursuing their interaction. During the revision of this manuscript, a study to identify the interactome of CCP6 using a Biotin-ID method confirmed the proximate location between CP110 and CCP6 [42]. It is possible that CCP6 is a part of a complex binding to CP110 which contributes to the stability of the protein at centrioles. Further investigation is required to address their possible interaction and functional relevance. Different from the other CCP members, Nna1/CCP1 and CCP4 possess a largely extended N-terminal domain, of which the exact function remains unknown [8, 43]. It is possible that this extended N-terminal domain masks their N-domain under certain circumstance and thereby regulates their interaction with the binding partners. Further experiments to verify this notion are ongoing.

CCP5 and CCP6 regulate the axonemal MT polyglutamylation in a way consistent with their substrate specificity. However, when their overexpression was induced after cilia formation, only CCP5 entered cilia, consistent with a previous observation [29], whereas CCP6 was only observed at the basal body (Fig. 8). It remains unclear how CCP6 affects tubulin modification in cilia. A recent study identified the mRNA of *Agbl4*, the gene coding CCP6, in cilia, suggesting that CCP6 could be locally translated [44]. It is possible that the level of locally synthesized CCP6 in cilia is too low to be detectable or it undergoes certain unidentified regulations. Our study supports the idea that the enzyme activity of CCP6 contributes to modification, as knocking down CCP6 increased the polyE signals in cilia, but not that of GT335 (Fig. 2). This also complies with the observation that co-depletion of CCP5 and CCP6 did not further increase the cilia length, as the persistence of long-chain modification due to loss of CCP6 can prevent CCP5 from further processing the branch point glutamate, leading to a reduction of CCP5 substrates in cilia.

## Conclusions

In summary, this work reveals a novel mechanism regulating ciliogenesis mediated by polyglutamylation erasers CCP5 and CCP6, and demonstrates their segregated functions in ciliogenesis and ciliary length control.

## Methods

### DNA constructs and siRNA

Plasmids for myc-tagged mouse CCPs are generous gifts from Dr. James I. Morgan at St. Jude Hospital [7, 10]. cDNA encoding human CCP5 and CCP6 were synthesized by General Biol (Anhui, China). Mouse or human CCP1, CCP4, and CCP6 were subcloned to pcDNA3.1( +) vector with fusion of a GFP tag at their N-termini, while human and mouse CCP5 were subcloned into pCMV-AC-GFP vector as we found that the proteins with GFP tag at their N-termini lost enzyme activity. Myc-tagged LacZ, CCP5, CCP6, or CCP5 truncated variants were amplified by PCR and subcloned into pcDNA3.1 zeo ( +) vector using HindIII & NotI restriction sites for expression in mammalian cells or stable cell line construction. To express GFP-tagged CCP5 or CCP6 using lentivius, the human CCP5 or CCP6 was cloned in pLVX-TetOne-Puro vector using a seamless cloning kit (GenStar, Beijing, China). All constructs were verified by DNA sequencing.

### siRNA

siRNA duplexes and RNAi negative control were obtained from General Biol. The efficiency of siRNAs was verified by western blotting or real-time PCR. Sequences of siRNA targeting corresponding mRNAs are as follows and siCCP5 #1 and siCCP6 #2 were used in rescue experiments.

- siCCP5 #1: 5′-GCUGAAGCCUGGAAACAAA -3′;
- siCCP5 #2: 5′-GGGAGGAAUGCCAGGAAAA-3′;
- siCCP6 #1: 5′-UGACCGAGAAGAAGAUAUU-3′;
- siCCP6 #2: 5′- CAGGCAAUGAUAUGGGAAA-3′;

### Antibodies

The following primary antibodies were used: anti-CP110 (Proteintech, 2780–1-AP, 1:2000 for western blotting, 1:500 for immunostaining), anti-CEP97 (Proteintech, 22,050–1-AP, 1:1000), anti-turboGFP (Origene, TA150041, 1:2000 for western blotting or Proteintech, tbfms, 1:500 for immunostaining), anti-myc-tag (Invitrogen, MA1-21,316, 1:2000 for western blotting, 1:500 for immunostaining), anti-His-tag (Immunoway, YM3004, 1:2000); anti-acetylated α-tubulin (Sigma, T6793, 1:500), anti-γ-tubulin (Sigma, T6557, 1:500), EP1332Y (Abcam, ab52866, 1:4000), GT335 (Adipogen, AG-20B-0020, 1:4000 for western blotting, 1:500 for immunostaining), PolyE (Adipogen, AG-25B-0030, 1:4000 for western blotting, 1:500 for immunostaining), anti-ARL13B (Proteintech, 17,711–1-AP, 1:500), anti-β-actin (Sungene biotech, KR9001T, 1:4000). The secondary antibodies were goat-anti-mouse or donkey-anti-rabbit antibodies coupled with horseadish peroxidase (Bioss, bs-40296G-HRP and bs-0295D-HRP 1:4000) for western blotting or those coupled with Alexa Fluor 488 or 568 for immunofluorescence analysis (Invitrogen, A32723 and A-11011 1:750).

### Cell culture, transfection, and ciliation induction

HEK293T cells were cultured in DMEM (Corning) supplemented with 10% FBS (Newzerum, Christchurch, New Zealand) and 1% penicillin/streptomycin at 37 °C with 5% CO_2_. hTERT-RPE-1 cells were maintained in DMEM/F12 (SparkJade, Xi’an, China) containing 10% FBS (Newzerum) and 0.01 mg/mL hygromycin B at 37 °C with 5% CO_2_. Plasmids and siRNAs were transfected into hTERT-RPE-1 or HEK293T cells using Fugene 6 (Promega, Madison, USA) or Lipofectamine™ 2000 (Invitrogen, Massachusetts, USA) according to the manufacturer’s protocols. For ciliation induction, cells at about 70% confluence were changed to respective medium without serum to induce cilia formation.

### Stable cell line construction

pcDNA3.1 zeo ( +) vector harboring myc-LacZ, CCP5, CCP6, or CCP5 truncated variants were transfected into HEK293T cells using Fugene 6 (Promega). The passages of cells were subjected to a selection with 400 μg/mL zeocin in the medium for 2 weeks. Subsequently, single clones of the cells were chosen and cultured in the medium containing 50 μg/mL zeocin for further immunoblotting and immunostaining analysis or transfection.

### RNA isolation and qRT-PCR

Trizol reagent (Invitrogen) was used to extract total RNA from cultured cells according to the manufacturer’s protocol. cDNA was reverse-transcribed using ABscript II cDNA First Strand Synthesis Kit (ABClonal, RK20400, Wuhan, China). Quantitative real-time PCR was performed using UltraSYBR Mixture (CWBIO, CW2602, Beijing, China) in a Roche LightCycler®96 System (Roche, Basel, Switzerland). CCP5 was amplified using primers 5′-TTCCAAAAGGGGCTGCTTCA-3′ and 5′-ACTGGCCATCTCTACGGTCT-3′ and β-actin was amplified using primers 5′-AACTGGGACGACATGGAGAAAA-3′ and 5′-GGATAGCACAGCCTGGATAGCA-3′. CCP5 RNA levels were quantified using the ΔΔCt (Livak) method and normalized to β-actin levels.

### Immunofluorescence microscopy

For immunofluorescence, cells cultured on coverslips were briefly washed by PBS once, followed by fixation in cold methanol at − 20 °C for 10 min or in 4% paraformaldehyde (PFA) in PBS at 37 °C for 15 min. After washed with PBS, cells were permeabilized with PBS containing 0.5% Triton X-100 and 2% bovine serum albumin (BSA, Genview, Florid, USA) or 10% horse serum for 1 h. Cells were then incubated with the primary antibody diluted in PBS containing 1% BSA or 1% horse serum at 4 °C overnight followed by incubation with Alexa Fluor coupled secondary antibody diluted in PBS containing 1% BSA or 1% horse serum at room temperature for 90 min. Cells were stained with DAPI to visualize the nucleus before slides were mounted with Fluoromount-G anti-fade mounting medium (SounthernBiotech, 0100–35, Alabama, USA).

All the samples were observed at room temperature under a fluorescence microscope (ECLIPSE 80i; Nikon, Tokyo, Japan) equipped with a 40 × 0.75 NA objective lens (Nikon) or a confocal microscope (TCS SP8; Leica, Wetzlar, Germany) equipped with 63 × 1.4 NA and 100 × 1.4 NA objective lens (Leica). For imaging of the colocalization of CCP5-GFP with γ-tubulin or CP110, the HyD detector and Lightning process were used. Images were acquired using NIS-Elements software (Nikon) or Las X software (Leica). Image processing was performed using ImageJ and Photoshop (Adobe, California, USA).

### Protein electrophoresis and immunoblotting

Proteins were resolved by SDS-PAGE gels using Mini-PROTEAN electrophoresis apparatus (BIORAD, California, USA) and transferred onto a nitrocellulose membrane (GE Healthcare, Pennsylvania, USA) using semi-dry transfer device (GE Healthcare, Pennsylvania, USA). Membranes were incubated with primary antibodies overnight at 4 °C and then HRP-conjugated secondary antibodies for 2 h at room temperature. The immunoreactivity of proteins was visualized with Western Bright ECL (Advansta, K-12043-D10, California, USA) using a Western Blot Imager (Vilber, Paris, France).

### Immunoprecipitation assay

HEK293T cells expressing myc-tagged LacZ, CCP5, CCP6, or CCP5 truncated variants were collected after wash with cold D-PBS (Solarbio, D1040, Beijing, China) and then lysed on ice with buffer-1 (25 mM Tris, 150 mM NaCl, 1 mM EDTA, 1% NP-40, 5% glycerol, pH7.4), supplemented with protease inhibitors (Meilun, Shanghai, China). The lysates were centrifuged at 13,000 × *g* for 10 min at 4 °C, and the supernatants were incubated with the Pierce Anti-c-Myc Magnetic Beads (Thermo Fisher Scientific, #88,844, Massachusetts, USA) at 4 °C overnight. After 3 times wash with buffer-2 (25 mM Tris, 150 mM NaCl, 0.05% Tween-20; pH7.5) and an additional wash with ultrapure water, the beads were boiled in Lane Marker Sample Buffer (Thermo Fisher Scientific, Massachusetts, USA) at 95 °C for 10 min to elute the binding proteins.

To immunoprecipitate CP110 interacting proteins, CP110 antibody was incubated with BeaverBeads™ Protein A/G Magnetic beads (Beaver, Guangzhou, China) with a rotation at a rate of 15 rpm for 1 h at RT. After extensive washing with PBS, the beads were incubated with cell lysates at 4 °C for at least 2 h and then washed with buffer-2 as described above. The beads were then boiled in SDS-PAGE loading buffer at 95 °C for 5 min. The eluted proteins were detected by immunoblotting as described above.

### Purification of GST-tagged protein

Mouse origin CCP5 with His-tag and GST-tag was cloned into pFastBac Dual vector and expressed in baculovirus expression system. Bacmid extracted from DH10bac competent cells was transfected into Sf9 cells by Cellfectin™ II Reagent (Gibco, #10,362,100, Massachusetts, USA). After 3 passages, the viral stock with validated expression was used to infect Sf9 cells for 72 h. For purification, cells were collected and lysed in the lysis buffer (250 mM KCl, 25 μg/mL DNase, 25 μg/mL lysozyme, 1 mM PMSF and protease inhibitor in 10 mM HEPES, pH 7.4) by sonication. After centrifugation at 15,000 × *g* for 30 min at 4 °C, supernatant was collected and incubated with Ni-Charged Resin (GenScript, L00666, Jiangsu, China) at 4° for 2 h. After washing with wash buffer (250 mM imidazole and 250 mM KCl in 10 mM HEPES, pH 7.4), His-GST-CCP5 was eluted with elution buffer (500 mM imidazole and 250 mM KCl in 10 mM HEPES, pH 7.4). The purity of eluted protein was analyzed by Western blotting and Coomassie Brilliant Blue staining after SDS-PAGE.

### GST pull-down assay

For pull-down assays, purified His-GST-CCP5 from Sf9 cells or His-GST protein was mixed with glutathione resin (GenScript, L00206, Jiangsu, China) and incubated at 4 °C for 2 h. After extensive washing with wash buffer (250 mM KCl in 10 mM HEPES, pH 7.4), the resins were incubated with supernatants from HEK293T cell lysates at 4 °C for 4 h and then washed with wash buffer to remove the unspecific bound proteins. The proteins bound with beads were eluted with elution buffer (250 mM KCl in 10 mM HEPES containing 10 mM reduced glutathione, pH 7.4) then boiled at 95 °C for 10 min in 5 × SDS loading buffer.

### Sample preparations for mass spectrometry analysis

Proteins immunoprecipitated from cells lines stably expressing myc-LacZ or myc-CCP6 using Pierce Anti-c-Myc Magnetic Beads were separated by SDS-PAGE and then subjected to silver staining (Beyotime, P0017S, Shanghai, China) according to the manufacturer’s protocol. The region of the gel between ~ 75 and 200 kDa that includes some distinctive protein bands in the myc-CCP6 expressing sample was excised from both samples and subjected to tryptic digestion according to a standard protocol [45]. The resulting peptides were analyzed using Orbitrap Fusion LUMOS ETD (Thermo Fisher Scientific, Massachusetts, USA) equipped with EASY-nLC 1200 column (Thermo Fisher Scientific, Massachusetts, USA) to determine CCP6-interacting proteins.

### Lentivirus production and infection

Lentiviral particles were produced and used to infect hTERT-RPE-1 cells for inducible expression of CCP5 and CCP6. For lentivirus production, HEK293T cells that reached 80% confluence were transfected with the lentiviral expression plasmids harboring CCP5 or CCP6, packaging plasmid (pAX2) and envelope plasmid (VSV-G) at a ratio of 5:3:3 for 72 h using Lipofectamine™ 2000. After 72 h, the medium containing virus particles was obtained after centrifuged and passed through a sterile 0.45-μm filter membrane to obtain infectible virus.

For viral infections, 400 μL of virus-containing medium was added to cells grown on coverslip in a 24-well plate containing 400 μL of complete medium in each well. Six hours after the first infection, 400 μL of virus was re-added. After another 12 h, the medium was changed to complete medium. To induce ciliogenesis, the medium was changed to serum-free medium 48 h after the first infection. To induce protein expression before serum starvation, doxycycline (Dox, 500 ng/mL) was added right after infection, while to induce CCP expression after serum starvation, Dox was added 48 h after infection when serum starvation started. All slides were harvested 48 h after serum starvation and fixed for the subsequent analysis.

### In vitro deglutamylation assay

In vitro deglutamylase activity assay was conducted using porcine tubulin as substrate as described previously [46]. Briefly, HEK293T cells transfected with Nna1/CCP1, CCP4, 5 or CCP6 were lysed in PBS containing 0.2% NP-40. After centrifugation at 13,000 × *g* at 4 °C for 10 min, 40 μL supernatant was incubated with 2 μg porcine tubulin (Cytoskeleton Inc., #T240-A, USA) at 37 °C for 3 h. Polyglutamylation levels were monitored by immunoblotting with the polyE and GT335 antibodies, and the tubulin levels were detected by EP1332Y antibody.

### Quantification of ciliation, cilia length, and immunoblotting band intensity

For ciliation and cilia length analysis, widefield images were taken as described above. The length of cilia or polyglutamylated axoneme was manually measured on images of cells stained with ARL13B (a cilia marker), GT335, or polyE respectively using the line segment tool in ImageJ. For the ciliogenesis assay, cells stained for the ciliary marker ARL13B from multiple field areas of 3 coverslips per condition were randomly selected and counted.

To quantify the intensity of immunoreactive signals of proteins on western blotting, gray scale analysis was performed using ImageJ or Evolution-Capt Edge (Vilber, Paris, France).

### Statistical analysis

Statistical analysis was performed using Prism 6 (GraphPad Software, California, USA). Statistical significance was determined by unpaired Student’s *t* test. *P* values < 0.05 were considered as statistically significant (*P < 0.05; **P < 0.01).

## Supplementary Information

**Additional file 1: Fig. S1.** The correlation of CCP5 and CCP6 expression with ciliation in HEK293T cells. **Fig. S2.** Validation of CCP5 and CCP6 siRNAs’ specificity and effects on glutamylation status in cells. **Fig. S3.** The spatial correlation between CCP5-GFP and CP110 at centrioles detected by IF. **Fig. S4.** Overexpression of respective mouse homologs suppressed cilia formation in CCP5 or CCP6 depleted cycling cells. **Fig. S5.** Comparison of CP110 and CEP97 levels in CCP5 and CCP6 individually or doubly depleted cells. **Fig. S6.** Validation of inducible expression of CCP5 and CCP6 in TetOne vector.

**Additional file 2:** **Table S1.** Original data of replicated experiments used for quantification.

**Additional file 3:** **Table S2.** Potential interaction proteins of CCP6 identified by CoIP-MS.

**Additional file 4.** Images of original blots for Figs. 1, 2, 3, 4, 5 and 6.

**Additional file 5.** Images of original blots for Additional file 1: Fig. S2-S6.

## Data Availability

All data generated or analyzed during this study are included in this published article and supplementary information files.

## Abbreviations

CCP: Cytosolic carboxypeptidase
CEP97: Centrosomal protein of 97 kDa
CP110: Centriolar coiled-coil protein of 110 kDa
Dox: Doxycycline
pcd: Purkinje cell degeneration
PTM: Posttranslational modification
qRT-PCR: Real-time quantitative polymerase chain reaction
SDS-PAGE: Sodium dodecyl sulfate–polyacrylamide gel electrophoresis
TTLL: Tubulin tyrosine ligase like
