# Supplementary material for: CCP5 and CCP6 retain CP110 and negatively regulate ciliogenesis
Source: BMC Biol. 2023 May 24;21:124. doi: 10.1186/s12915-023-01622-1 (PMC10210458; doi:10.1186/s12915-023-01622-1)
Supplement: Supplementary file 1 — Additional file 1: Fig. S1. The correlation of CCP5 and CCP6 expression with ciliation in HEK293T cells. Fig. S2. Validation of CCP5 and CCP6 siRNAs’ specificity and effects on glutamylation status in cells. Fig. S3. The spatial correlation between CCP5-GFP and CP110 at centrioles detected by IF. Fig. S4. Overexpression of respective mouse homologs suppressed cilia formation in CCP5 or CCP6 depleted cycling cells. Fig. S5. Comparison of CP110 and CEP97 levels in CCP5 and CCP6 individually or doubly depleted cells. Fig. S6. Validation of inducible expression of CCP5 and CCP6 in TetOne vector. [file 12915_2023_1622_MOESM1_ESM.docx]

**SUPPLEMENTARY MATERIALS**

**CCP5 and CCP6 retain CP110 and negatively regulate ciliogenesis**

Yujuan Wang^1#^, Yuan Zhang^1#^, Xinyu Guo^1^, Yiqiang Zheng^1^, Xinjie Zhang^1^, Shanshan Feng^2^, and Hui-Yuan Wu^1^*

^1^School of Pharmaceutical Science and Technology, Tianjin University, Tianjin, 300072, China

^2^Key Laboratory of Regenerative Medicine, Ministry of Education, Department of Developmental and Regenerative Biology, Jinan University, Guangzhou, 51063, China

# Equal contribution

* Corresponding Author

Hui-Yuan Wu

School of Pharmaceutical Science and Technology,

Tianjin University,

92 Weijin Road, Building 24, Room 417-8

Tianjin, 300072, China

E-mail: [huiyuan.wu@tju.edu.cn](mailto:Huiyuan.wu@tju.edu.cn)

ORCID: 0000-0002-5586-8857

**
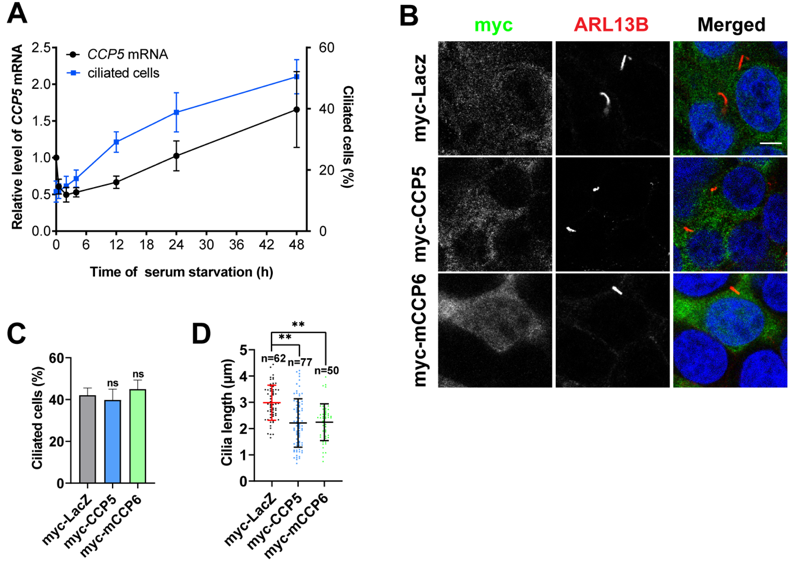
**

**Fig. S1. The correlation of CCP5 and CCP6 expression with ciliation in HEK293T cells**

**A)** The time course of endogenous CCP5 mRNA levels (black line) and the percentage of ciliated cells (blue line) in HEK293T cells after serum starvation. The expression level of CCP5 is dramatically reduced in 30 min after serum starvation and remains low after 4 h when ciliogenesis initiates, but gradually recovered to a level comparable to that before serum starvation at 48 h when cell ciliation is completed. The bars represent the mean ± s.d. from 3 independent experiments (Additional File 2). For the percentage of ciliated cells, at least 65 cells were analyzed per experimental condition (Additional File 2). **B)** After 24 h serum starvation, HEK293T cells stably expressed with myc-LacZ, myc-CCP5 or myc-mCCP6 were stained with myc (green), ARL13B (red), and the nuclear is visualized by DAPI (blue) staining. **C,D)** The cells stably expressing myc-CCP5 or myc-mCCP6 cells can form cilia at a rate similar to the control (C), but the length of cilia was shorter (D). The quantification of the ciliated cells from 3 independent experiments with at least 50 cells analyzed per experimental condition in each experiment is shown in **C** and **D** (Additional File 2). Error bars represent s.d.. ∗∗, *P* < 0.01, Student’s t-test. Scale bars: 10 µm.


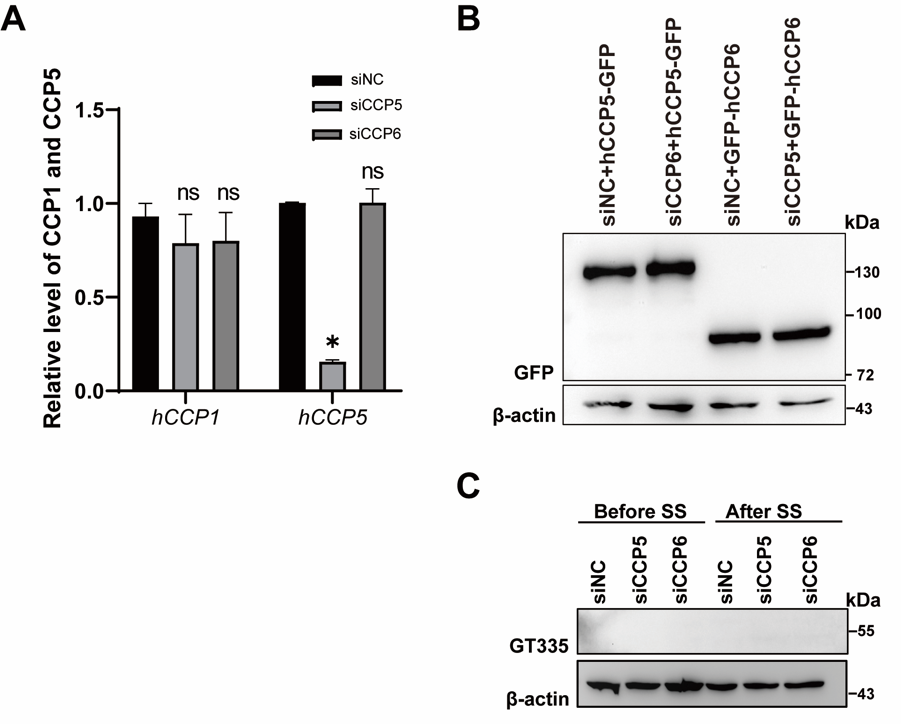


**Fig. S2. Validation of CCP5 and CCP6 siRNAs’ specificity and effects on glutamylation status in cells. A)** qRT-PCR analysis for the effects of siCCP6 on expression of Nna1/CCP1 or CCP5 in RPE-1 cells. The bars represent the mean ± s.e.m. from 3 independent experiments (Additional File2). **B)** Western blotting analysis of the expression level of CCP5 and CCP6 in corresponding stable cells after transfected with siCCP6 or siCCP5 respectively. **C)** The glutamylation levels of RPE-1 cells were monitored with the immunoreactivity for GT335 after transfected with siRNAs. The GT335 signals are hardly detectable in the control and siCCPs-transfected cells before or after ciliogenesis.


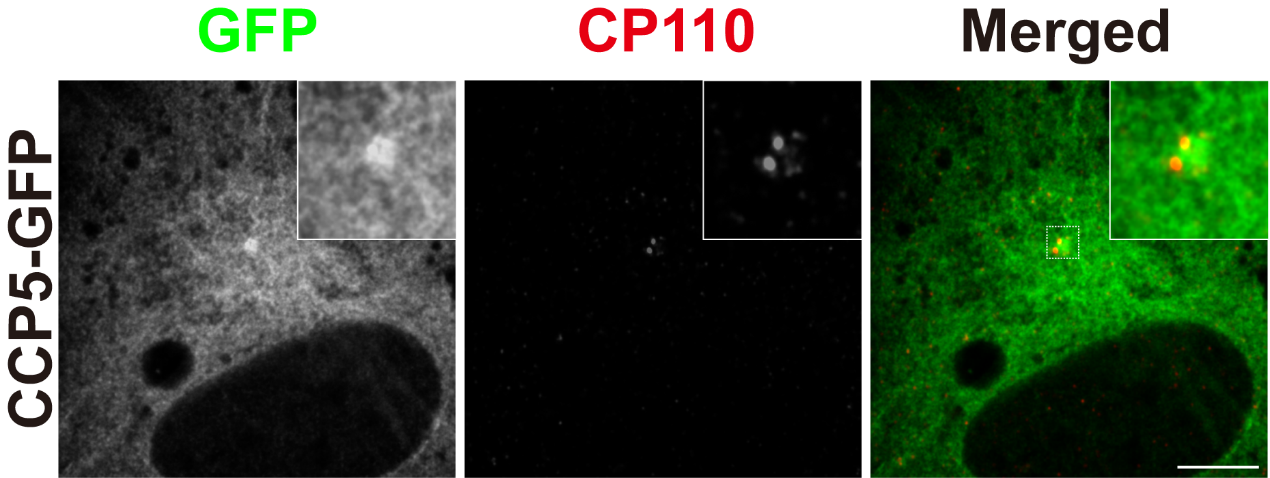


**Fig. S3. Immunofluorescence imaging to determine the spatial correlation between CCP5-GFP and CP110 at centrioles.** Representative immunofluorescence images showed that in hTERT-RPE1 cells transfected with CCP5-GFP, CCP5 (green) was partially co-localized with CP110 (red). The inserts are higher magnification views of the boxed region. Scale bar: 6 μm.


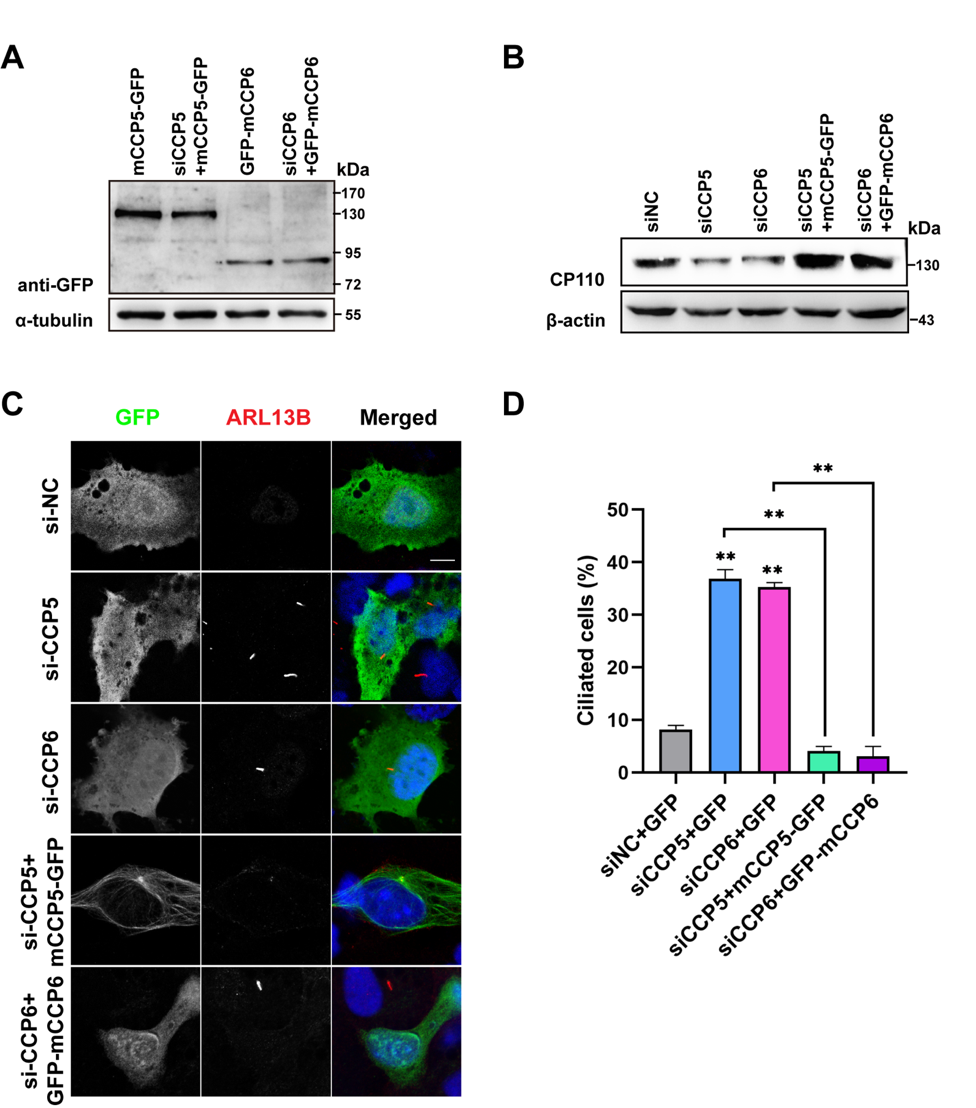


**Fig. S4. The abnormal cilia formation in CCP5 or CCP6 depleted cycling cells can be rescued by overexpression of their mouse homologs respectively. A)** siRNA of human origin *CCP5* and *CCP6* did not reduce protein levels of overexpressed mouse *CCP5* or *CCP6* in HEK293 cells respectively, allowing them being used for rescue experiments. **B)** The reduced CP110 protein levels in CCP5 or CCP6 depleted cells can be rescued by overexpression of mouse CCP5 and CCP6 respectively. **C)** Representative images of hTERT-RPE1 cells cultured in medium supplemented with serum that were transfected with indicated siRNAs and GFP, mouse CCP5 or CCP6 expressing plasmids and immunostained with GFP (green), ARL13B (red) with nuclei visualized with DAPI. **D)** Quantification of results presented in C showed that CCP5 or CCP6 depletion increased the ratio of ciliated cells from ~10% to >30%, while co-transfection of mouse CCP5 or CCP6 respectively reduced the ciliation ratios to the normal level. The bars represent the mean ± s.d. from 3 independent experiments (Additional File 2). For each experiment, at least 30 cells were counted. ∗∗, *P* < 0.01. Student’s *t*-test. Scale bars: 10 µm.

**
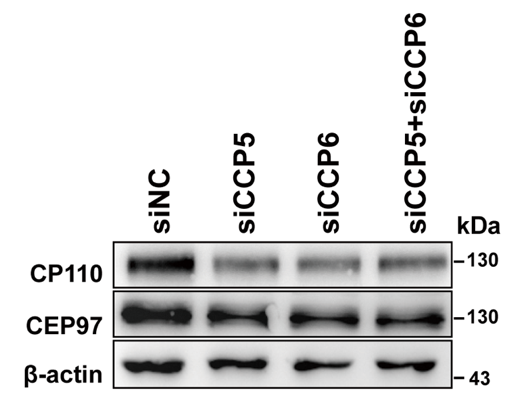
**

**Fig. S5. Comparison of CP110 and CEP97 levels in CCP5 and CCP6 individually or doubly depleted HEK cells.** The levels of CP110 and CEP97 in CCP5 and CCP6 co-depleting cells were not further reduced compared to that in CCP5 or CCP6 singly depleting cells.

**
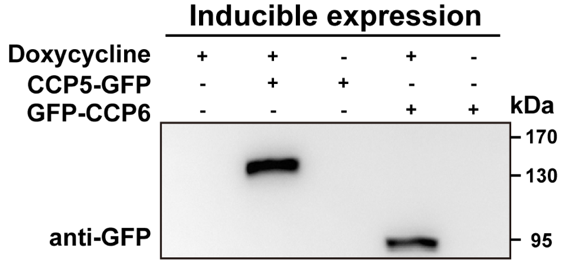
**

**Fig. S6. Validation of inducible expression of CCP5 and CCP6 in TetOne vector.** Lysates of HEK293T cells transfected with Tet-on controlled CCP5 or CCP6 expressing plasmid with or without doxycycline (Dox) addition were analyzed with anti-GFP via immunoblotting.
