# Supplementary material for: CCP5 and CCP6 retain CP110 and negatively regulate ciliogenesis
Source: BMC Biol. 2023 May 24;21:124. doi: 10.1186/s12915-023-01622-1 (PMC10210458; doi:10.1186/s12915-023-01622-1)
Supplement: Supplementary file 4 — Additional file 4. Images of original blots for Figs. 1, 2, 3, 4, 5 and 6. [file 12915_2023_1622_MOESM4_ESM.pptx]

## Slide 1
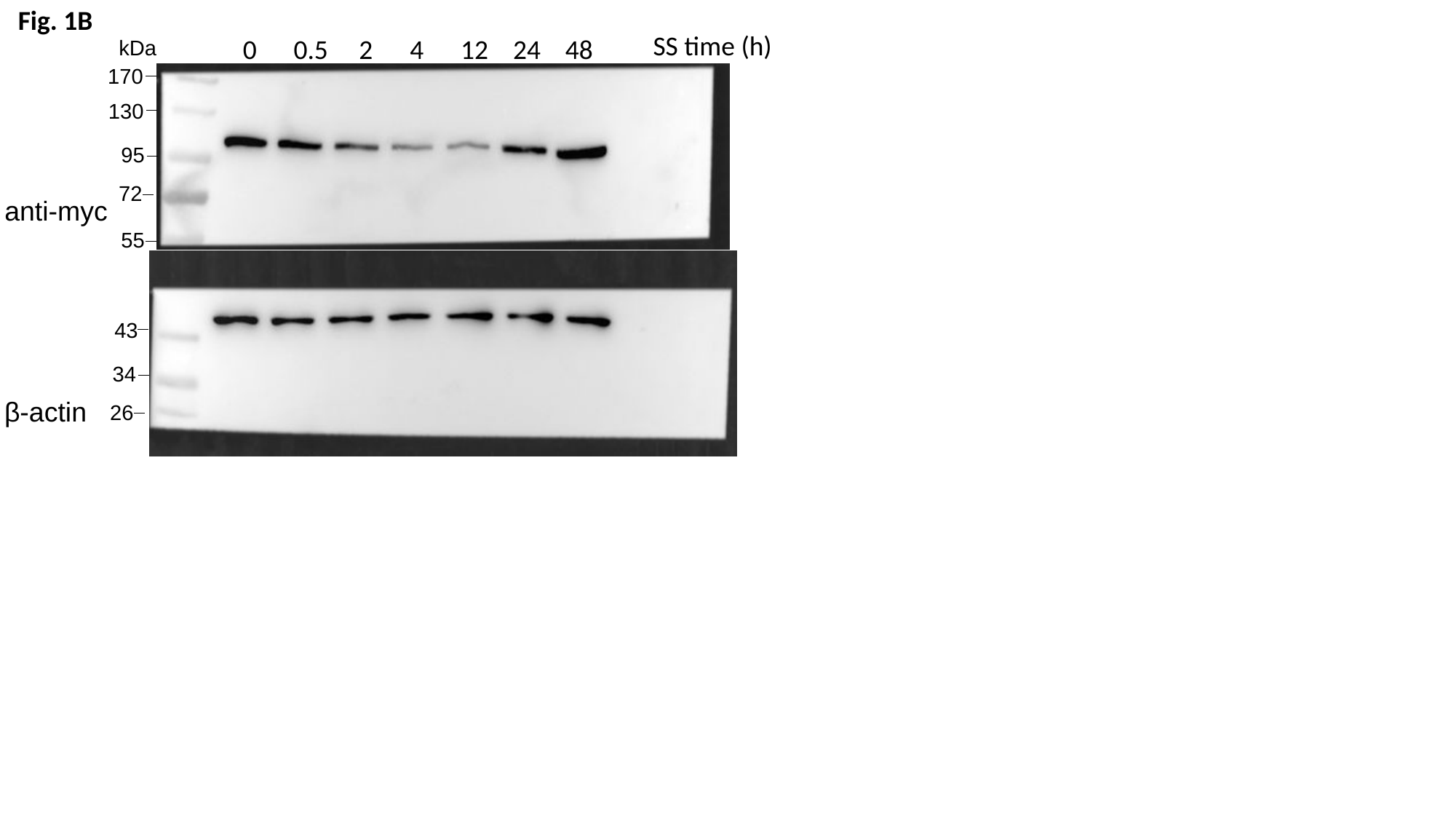

Fig. 1B
SS time (h)
0 0.5 2 4 12 24 48
kDa
170
130
95
72
anti-myc
55
43
34
β-actin
26

## Slide 2
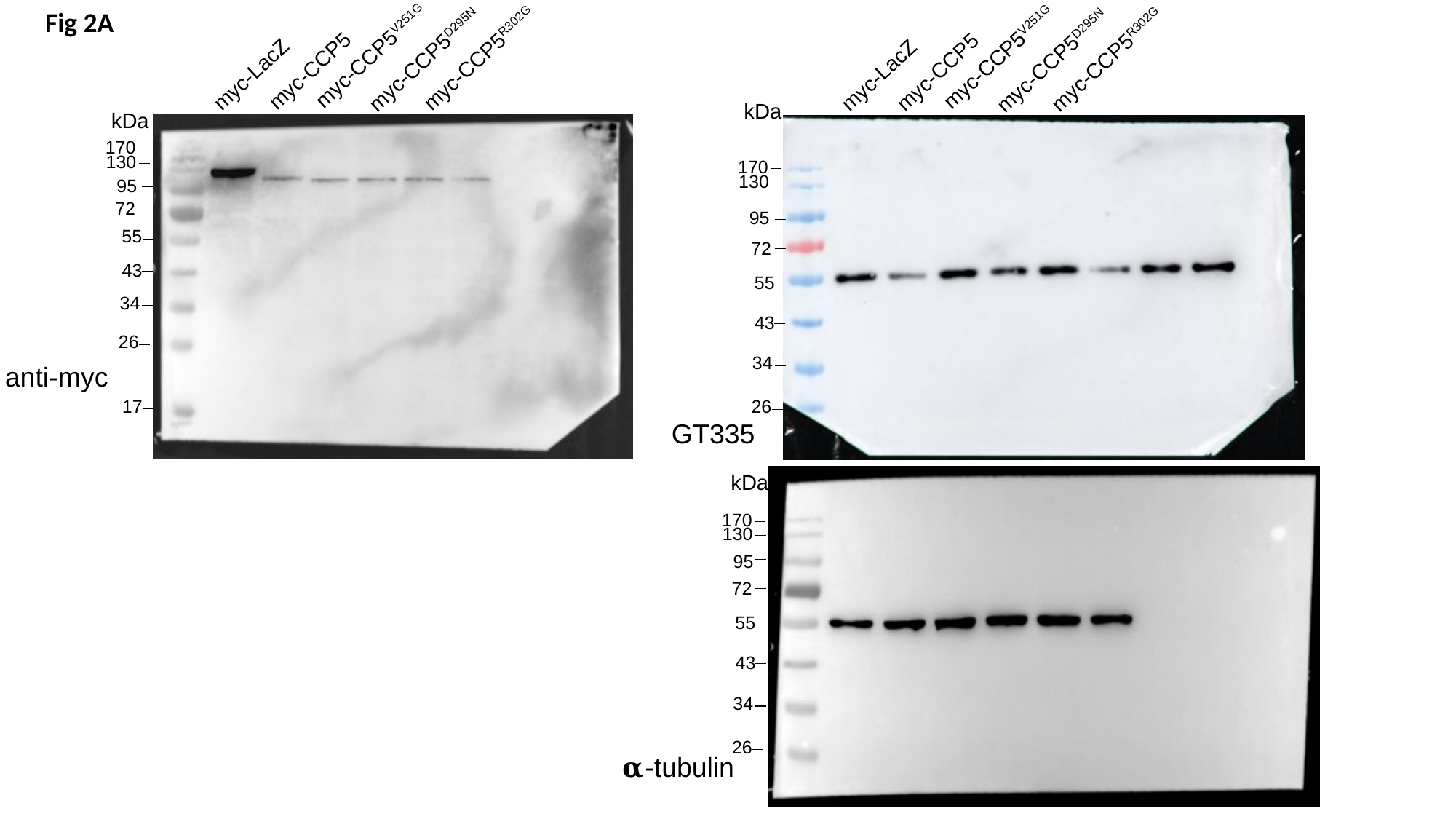

Fig 2A
myc-CCP5V251G
myc-CCP5V251G
myc-CCP5R302G
myc-CCP5R302G
myc-CCP5D295N
myc-CCP5D295N
myc-CCP5
myc-CCP5
myc-LacZ
myc-LacZ
kDa
kDa
170
130
170
130
95
72
95
55
72
43
55
34
43
26
34
anti-myc
17
26
GT335
kDa
170
130
95
72
55
43
34
26
𝛂-tubulin

## Slide 3
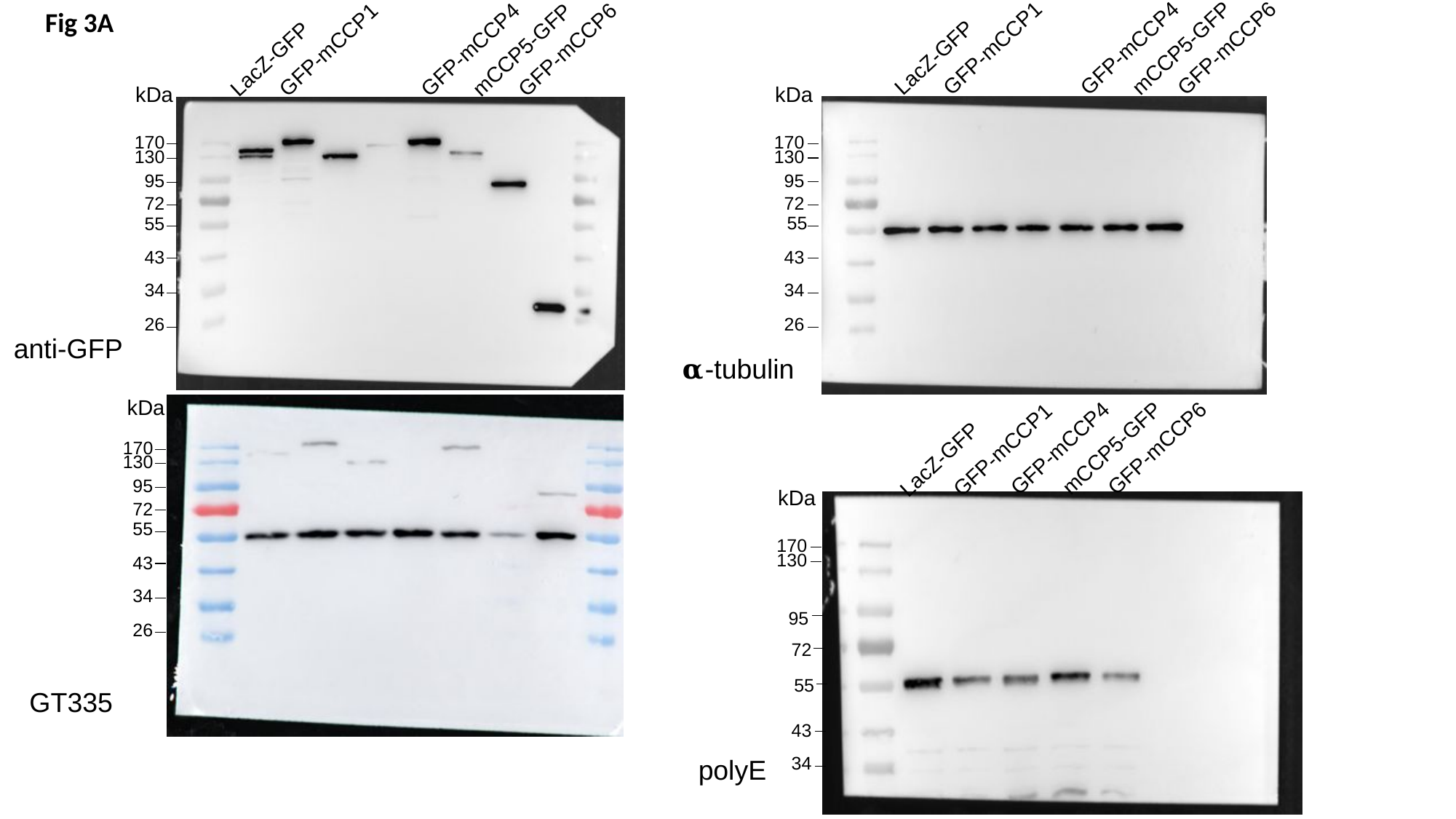

Fig 3A
GFP-mCCP1
GFP-mCCP4
mCCP5-GFP
GFP-mCCP6
GFP-mCCP1
GFP-mCCP4
mCCP5-GFP
GFP-mCCP6
LacZ-GFP
LacZ-GFP
kDa
kDa
170
170
130
130
95
95
72
72
55
55
43
43
34
34
26
26
anti-GFP
𝛂-tubulin
kDa
GFP-mCCP4
mCCP5-GFP
GFP-mCCP6
170
GFP-mCCP1
LacZ-GFP
130
95
kDa
72
55
170
130
43
34
95
26
72
55
GT335
43
34
polyE

## Slide 4
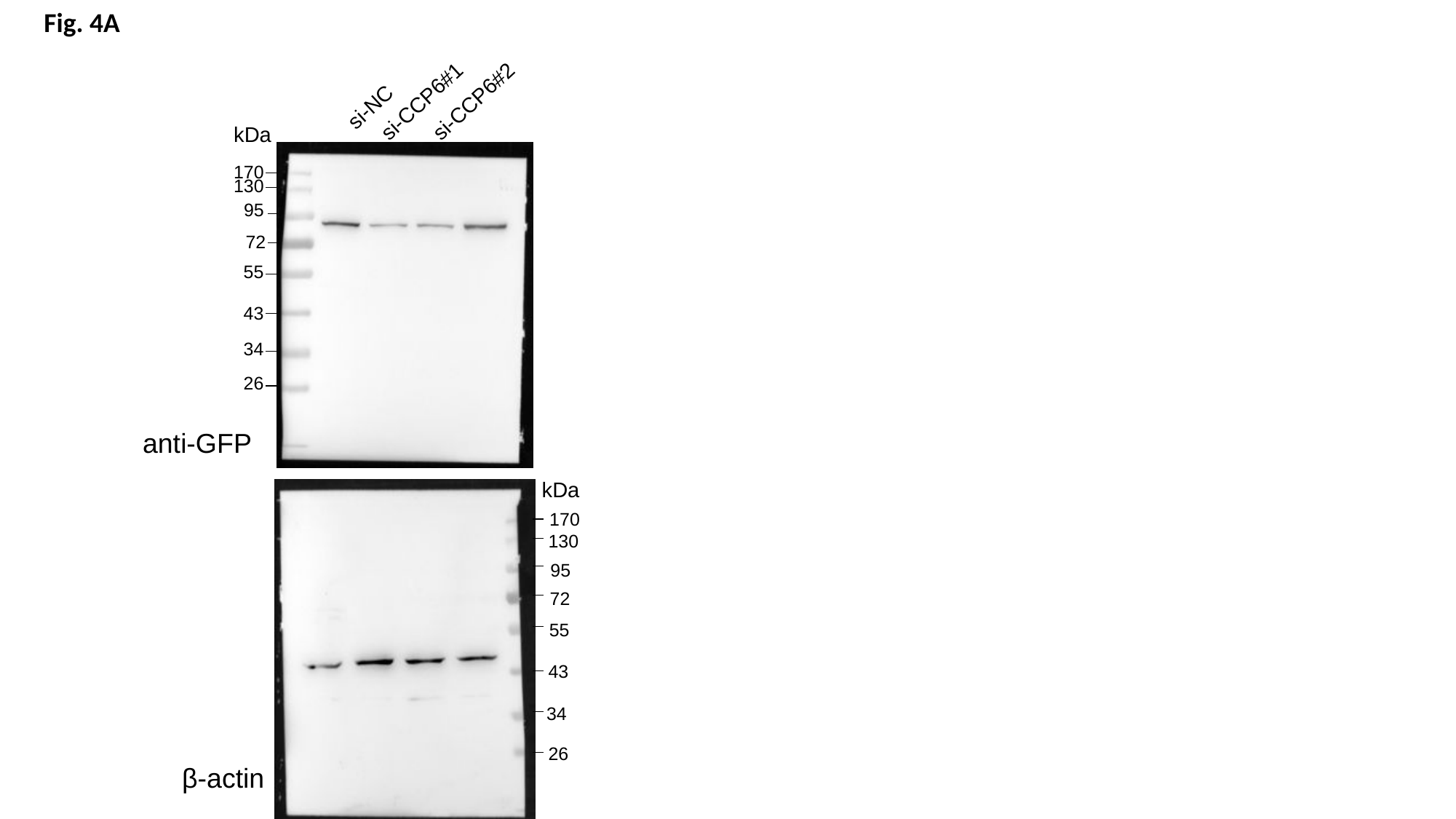

Fig. 4A
si-CCP6#1
si-CCP6#2
si-NC
kDa
170
130
95
72
55
43
34
26
anti-GFP
kDa
170
130
95
72
55
43
34
26
β-actin

## Slide 5
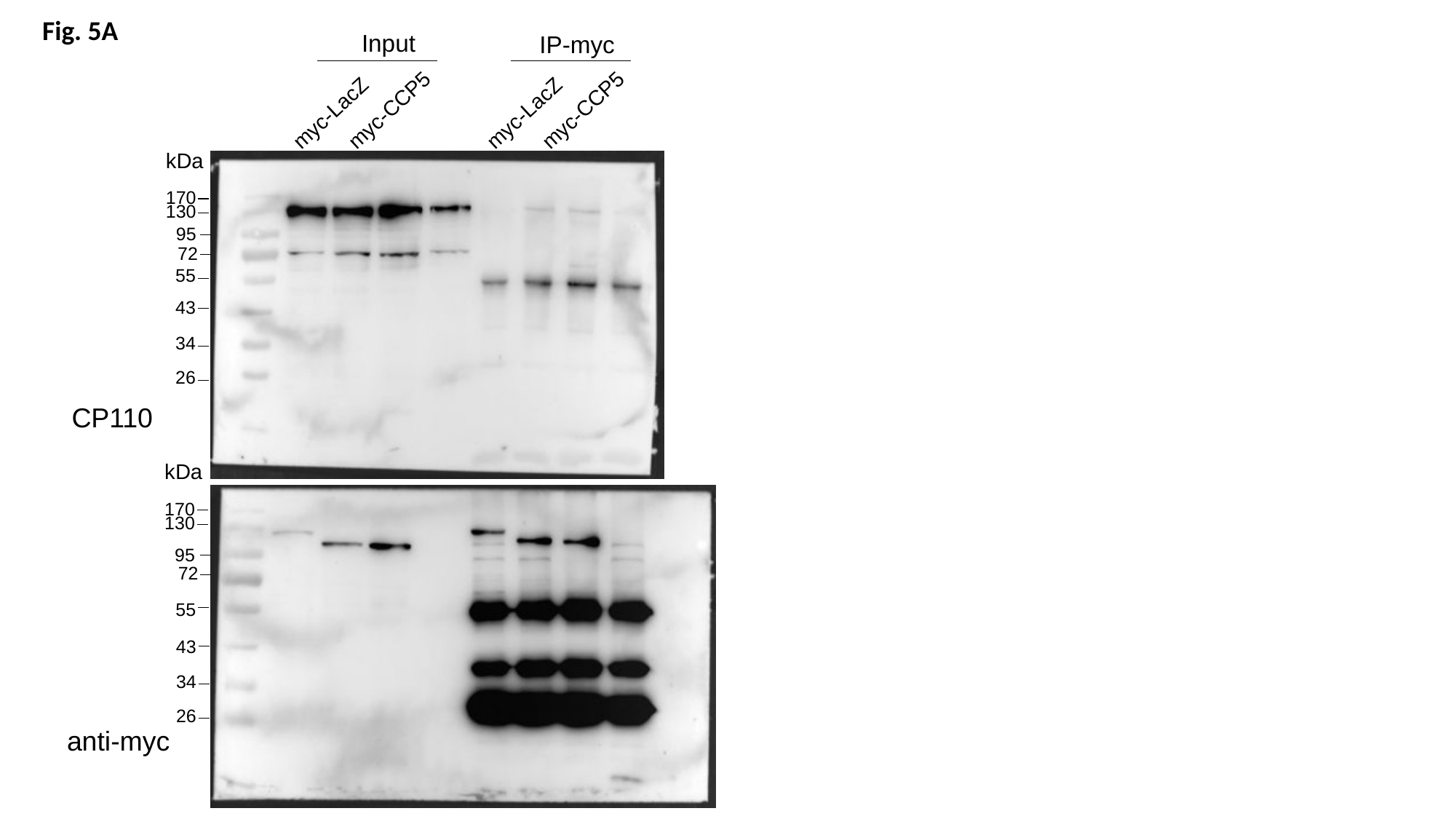

Fig. 5A
Input
IP-myc
myc-CCP5
myc-CCP5
myc-LacZ
myc-LacZ
kDa
170
130
95
72
55
43
34
26
CP110
kDa
170
130
95
72
55
43
34
26
anti-myc

## Slide 6
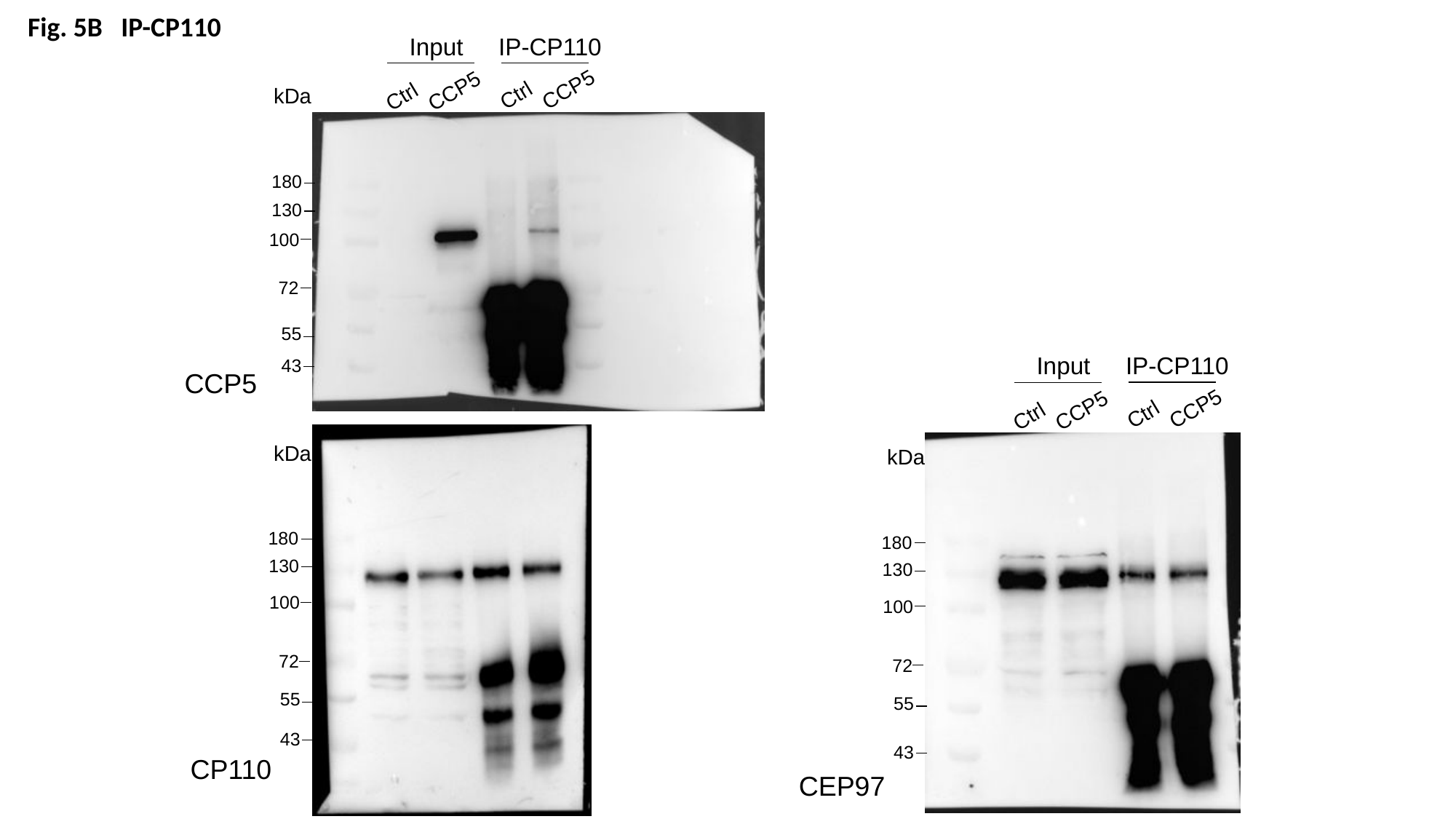

Fig. 5B IP-CP110
Input
IP-CP110
CCP5
CCP5
Ctrl
kDa
Ctrl
180
130
100
72
55
Input
IP-CP110
43
CCP5
CCP5
CCP5
Ctrl
Ctrl
kDa
kDa
180
180
130
130
100
100
72
72
55
55
43
43
CP110
CEP97

## Slide 7
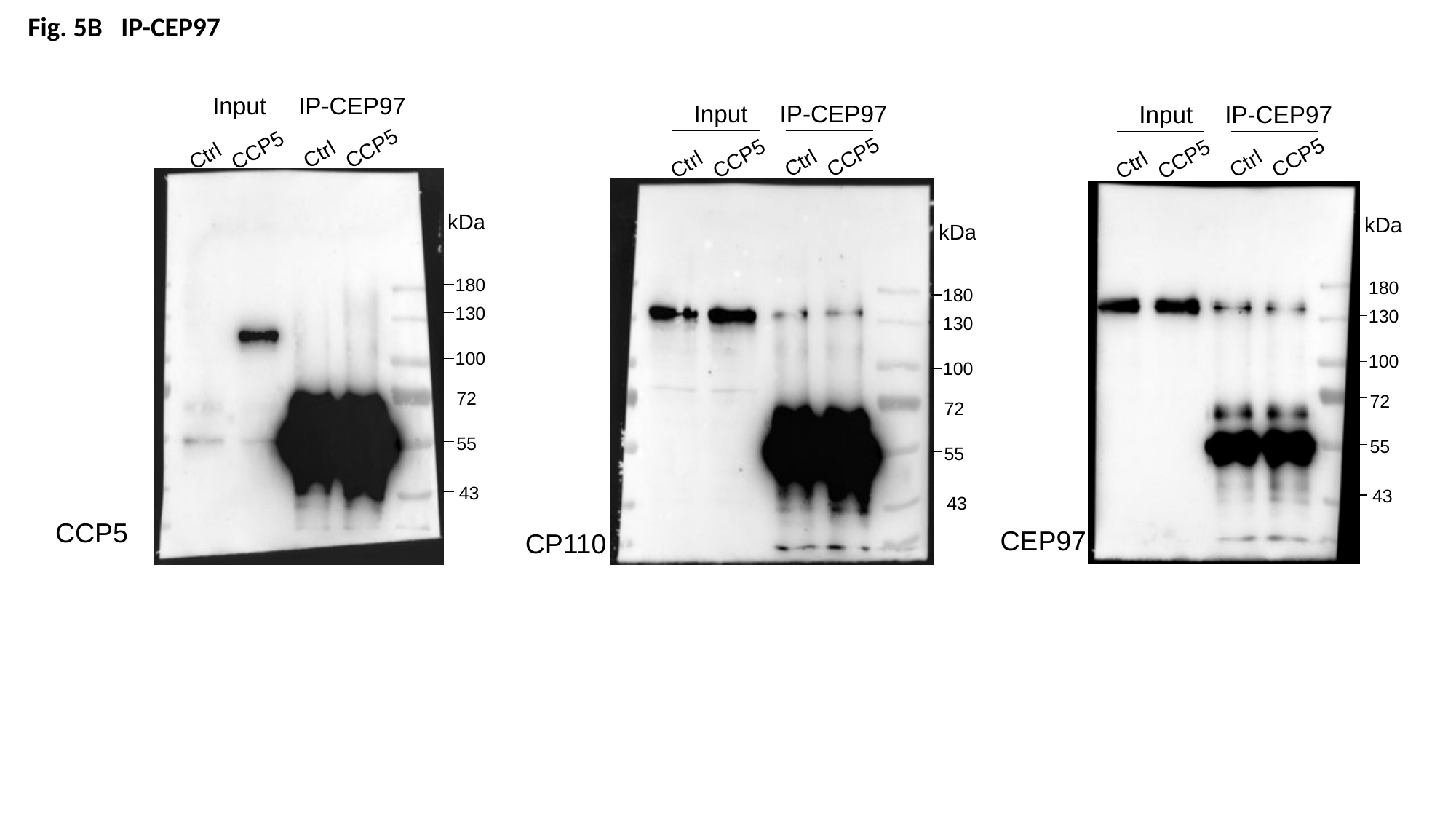

Fig. 5B IP-CEP97
Input
IP-CEP97
Input
IP-CEP97
Input
IP-CEP97
CCP5
CCP5
Ctrl
Ctrl
CCP5
CCP5
CCP5
CCP5
Ctrl
Ctrl
Ctrl
Ctrl
kDa
kDa
kDa
180
180
180
130
130
130
100
100
100
72
72
72
55
55
55
43
43
43
CCP5
CEP97
CP110

## Slide 8
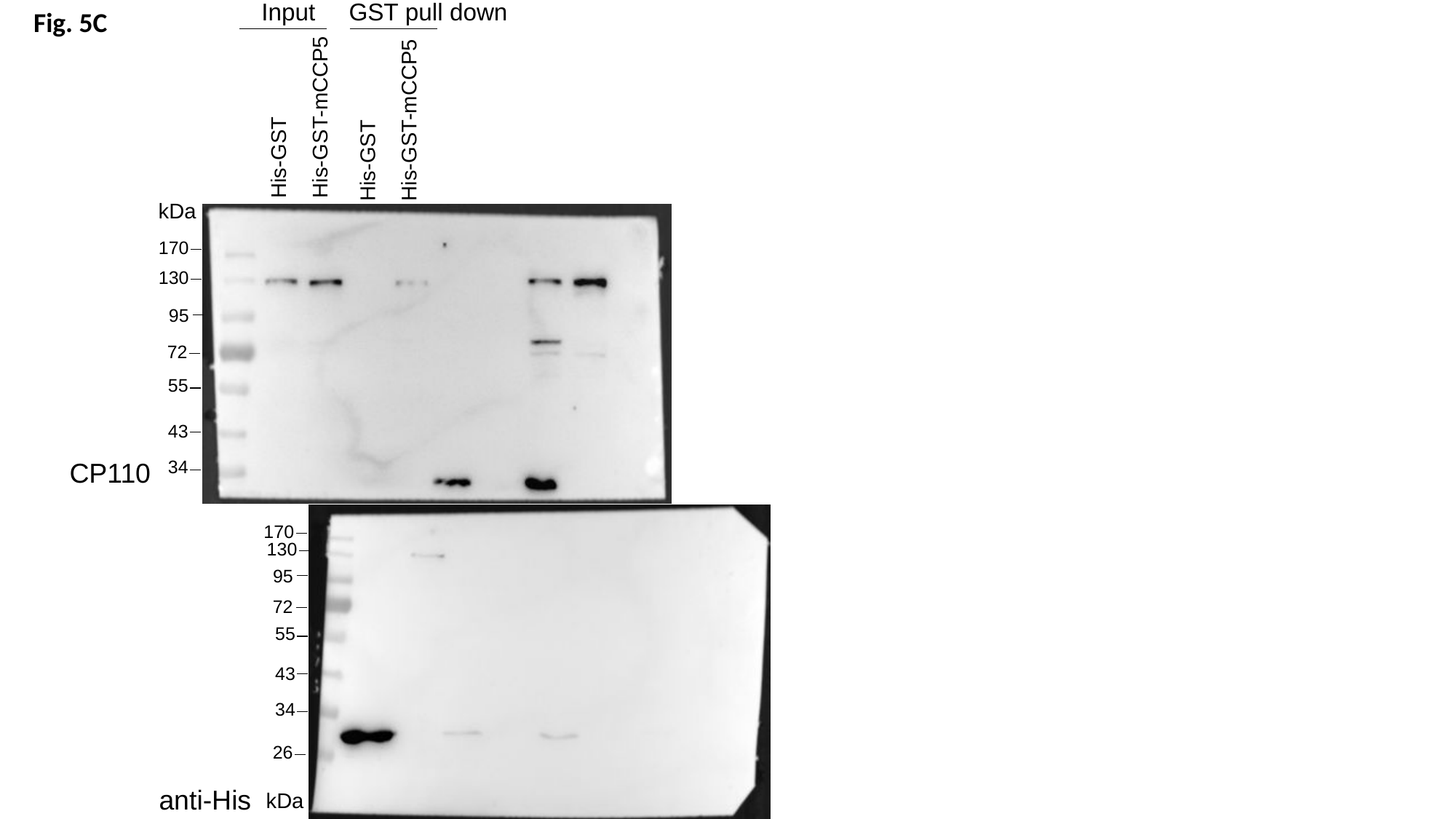

Fig. 5C
Input
GST pull down
His-GST-mCCP5
His-GST-mCCP5
His-GST
His-GST
kDa
170
130
95
72
55
43
CP110
34
170
130
95
72
55
43
34
26
anti-His
kDa

## Slide 9
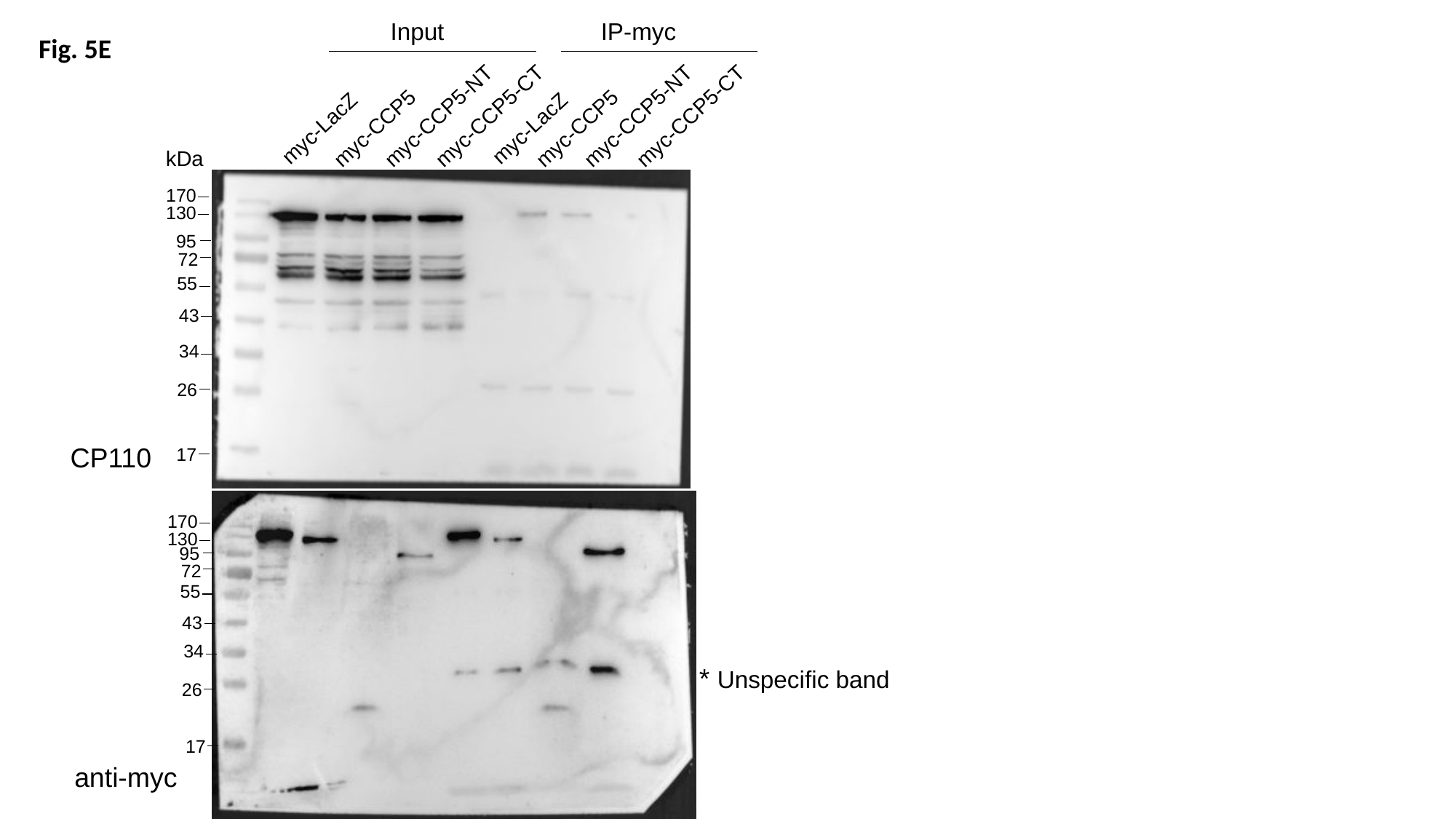

Input
IP-myc
Fig. 5E
myc-CCP5-CT
myc-CCP5-CT
myc-CCP5-NT
myc-CCP5-NT
myc-LacZ
myc-LacZ
myc-CCP5
myc-CCP5
kDa
170
130
95
72
55
43
34
26
CP110
17
170
130
95
72
55
43
34
* Unspecific band
26
17
anti-myc

## Slide 10
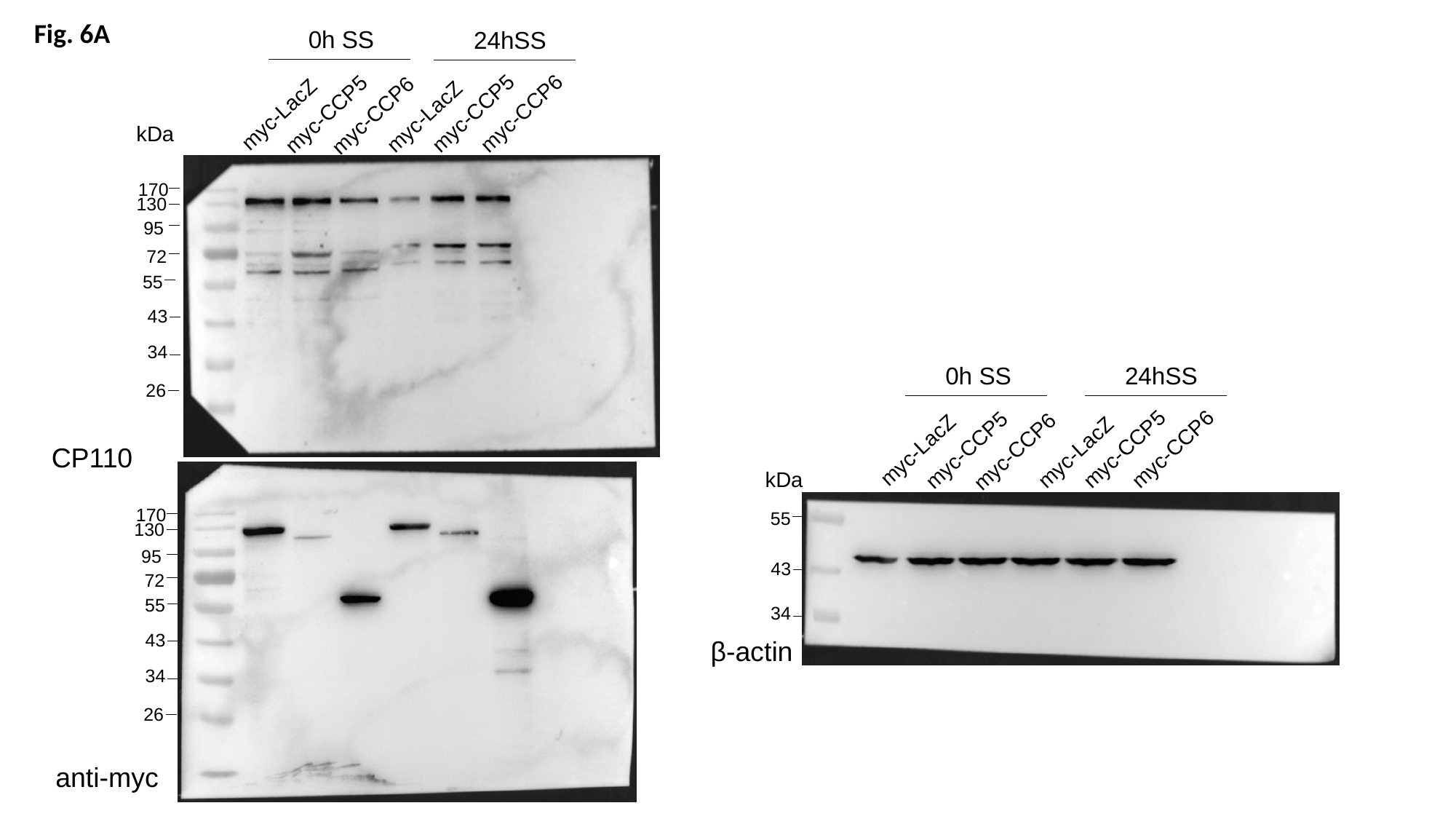

Fig. 6A
0h SS
24hSS
myc-CCP5
myc-CCP6
myc-LacZ
myc-CCP5
myc-CCP6
myc-LacZ
kDa
170
130
95
72
55
43
34
0h SS
24hSS
26
myc-CCP5
myc-CCP6
myc-LacZ
myc-CCP5
myc-CCP6
myc-LacZ
CP110
kDa
170
55
130
95
43
72
55
34
43
β-actin
34
26
anti-myc

## Slide 11
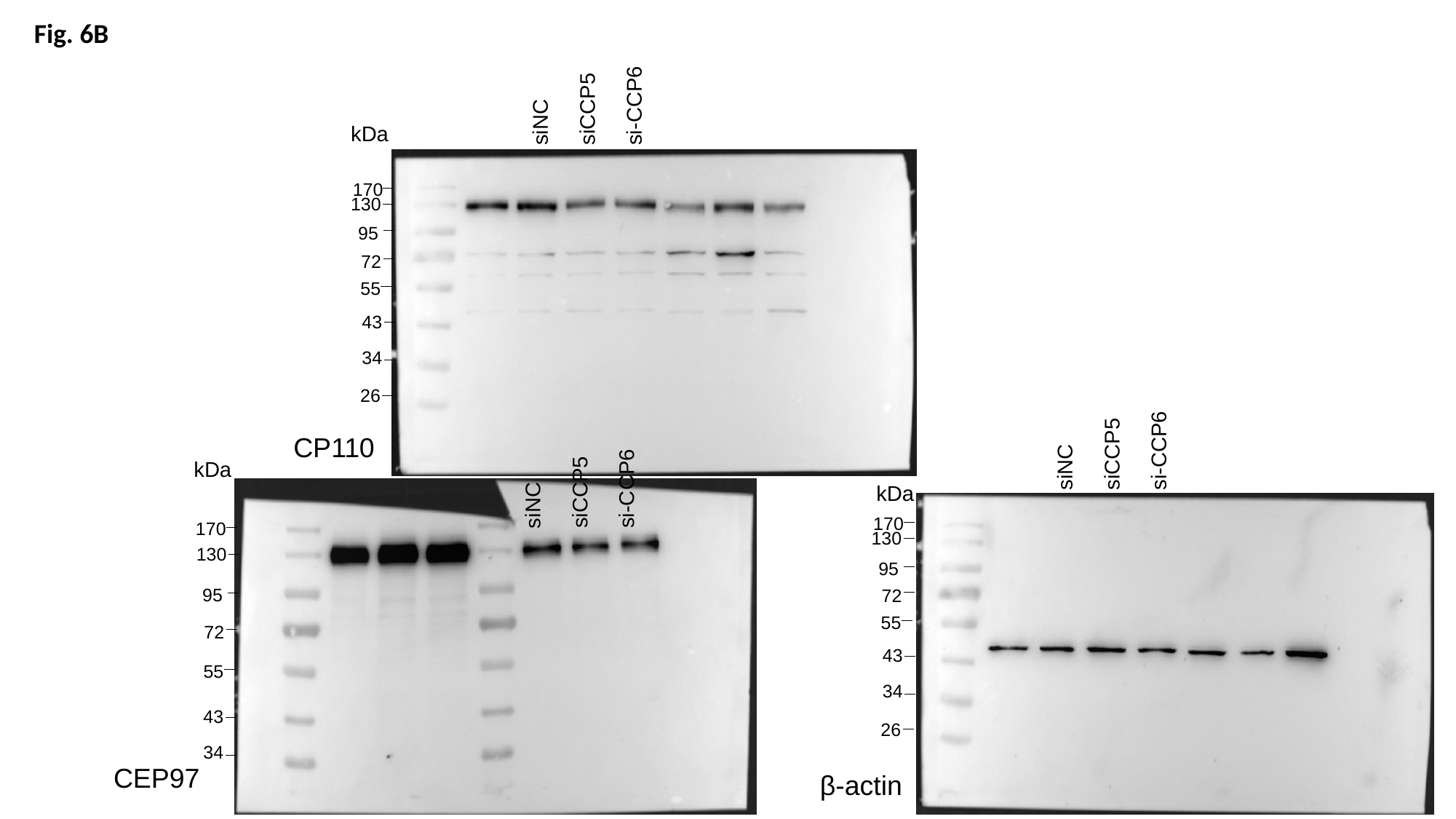

Fig. 6B
si-CCP6
siCCP5
siNC
kDa
170
130
95
72
55
43
34
26
CP110
si-CCP6
siCCP5
siNC
kDa
si-CCP6
siCCP5
kDa
siNC
170
170
130
130
95
95
72
55
72
43
55
34
43
26
34
CEP97
β-actin
