# Supplementary material for: CCP5 and CCP6 retain CP110 and negatively regulate ciliogenesis
Source: BMC Biol. 2023 May 24;21:124. doi: 10.1186/s12915-023-01622-1 (PMC10210458; doi:10.1186/s12915-023-01622-1)
Supplement: Supplementary file 5 — Additional file 5. Images of original blots for Additional file 1: Fig. S2-S6. [file 12915_2023_1622_MOESM5_ESM.pptx]

## Slide 1
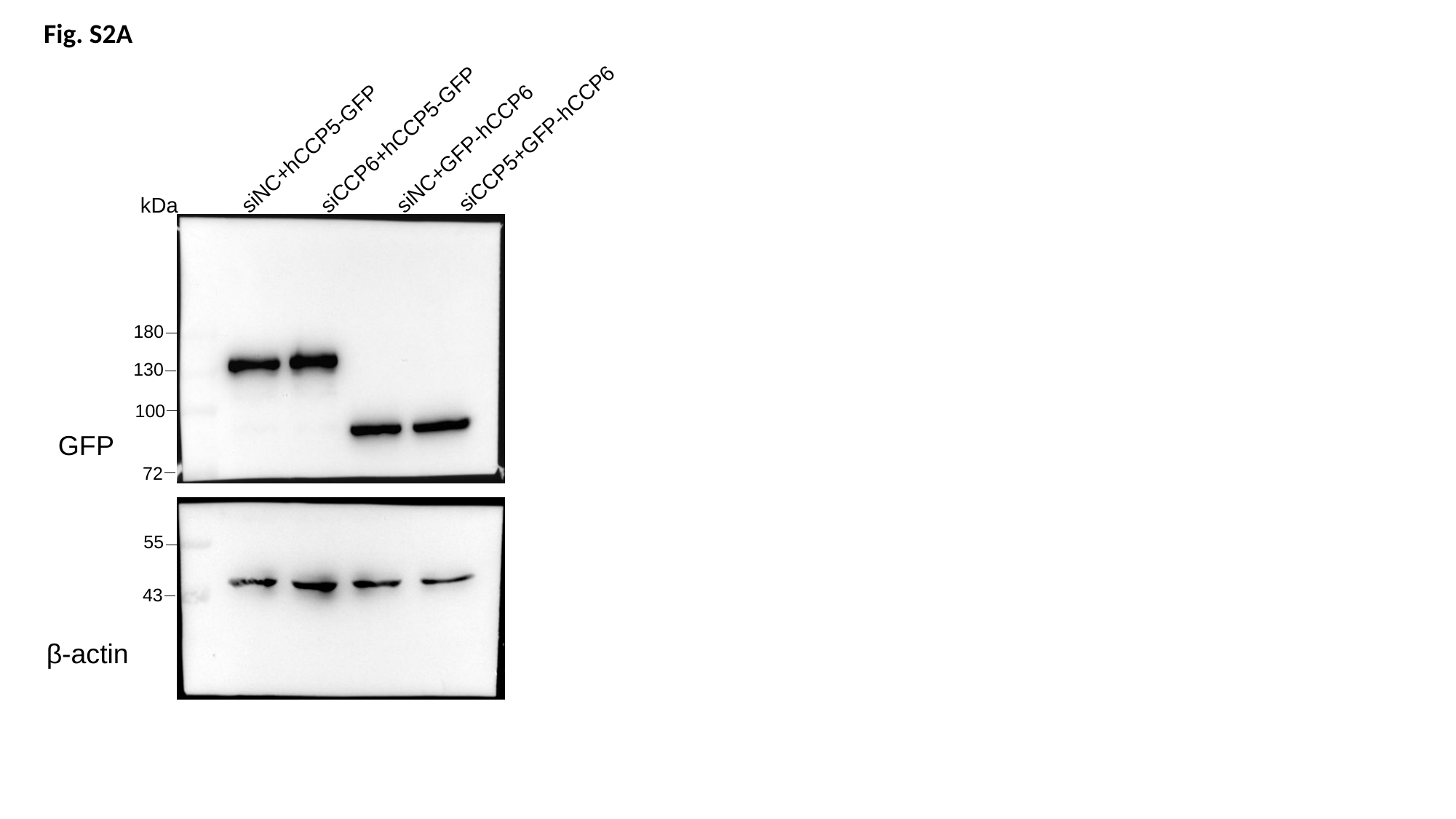

Fig. S2A
siCCP5+GFP-hCCP6
siCCP6+hCCP5-GFP
siNC+hCCP5-GFP
siNC+GFP-hCCP6
kDa
180
130
100
GFP
72
55
43
β-actin

## Slide 2
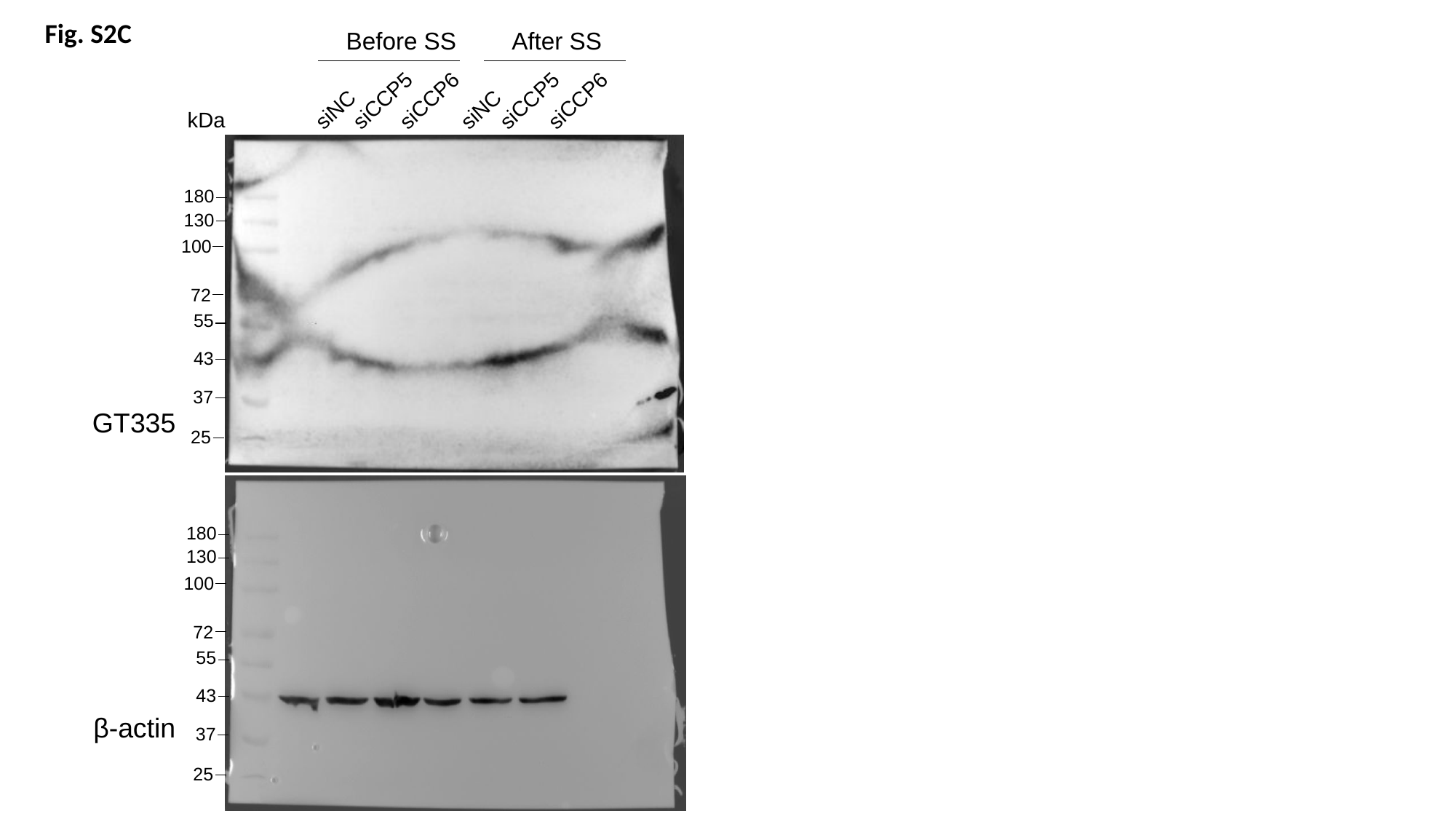

Fig. S2C
Before SS
After SS
siCCP5
siCCP6
siCCP5
siCCP6
siNC
siNC
kDa
180
130
100
72
55
43
37
GT335
25
180
130
100
72
55
43
β-actin
37
25

## Slide 3
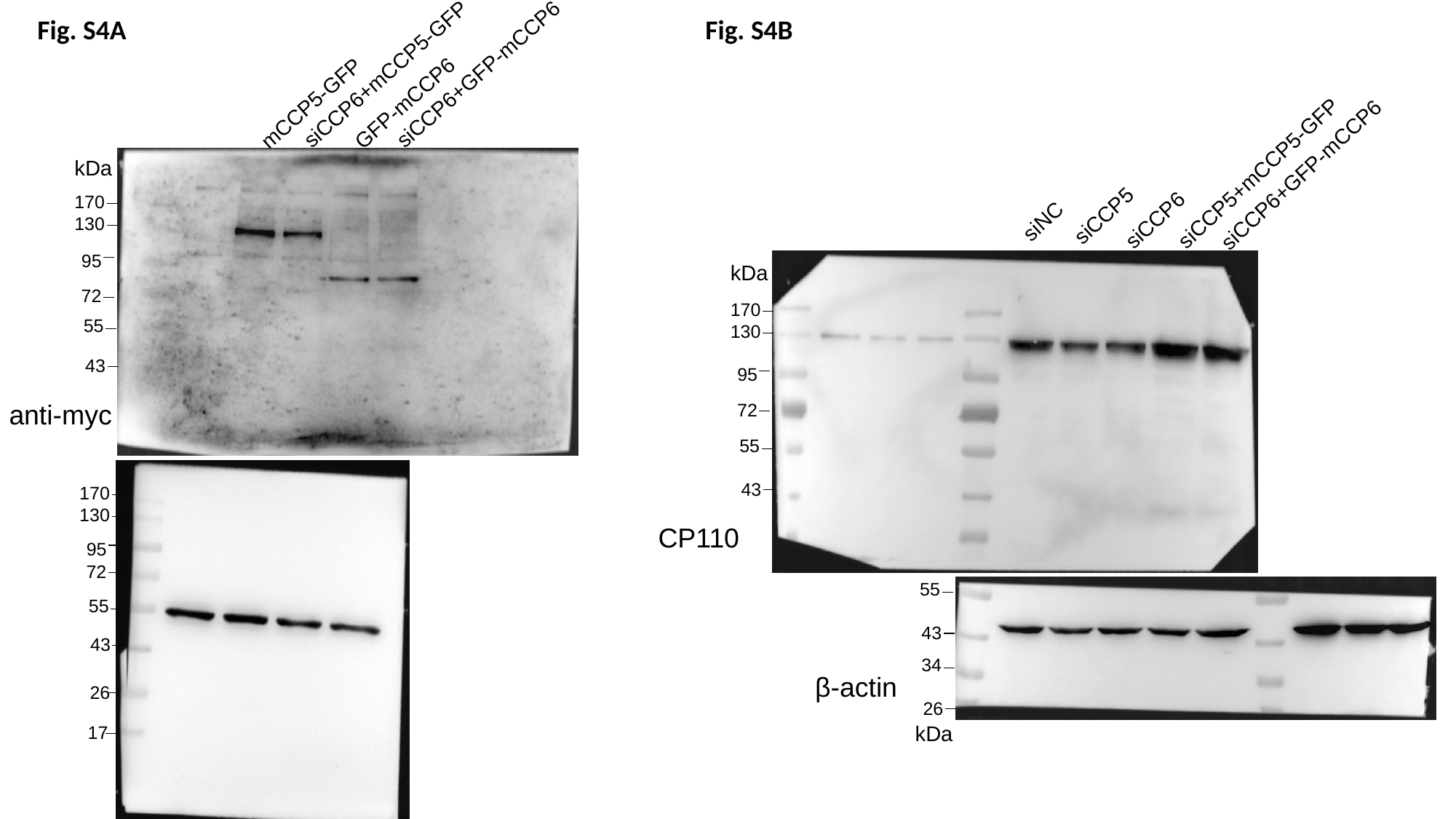

Fig. S4A
Fig. S4B
siCCP6+mCCP5-GFP
siCCP6+GFP-mCCP6
mCCP5-GFP
GFP-mCCP6
kDa
siCCP5+mCCP5-GFP
siCCP6+GFP-mCCP6
170
siCCP5
siCCP6
siNC
130
95
kDa
72
170
55
130
43
95
anti-myc
72
55
43
170
130
CP110
95
72
55
55
43
43
34
β-actin
26
26
kDa
17

## Slide 4
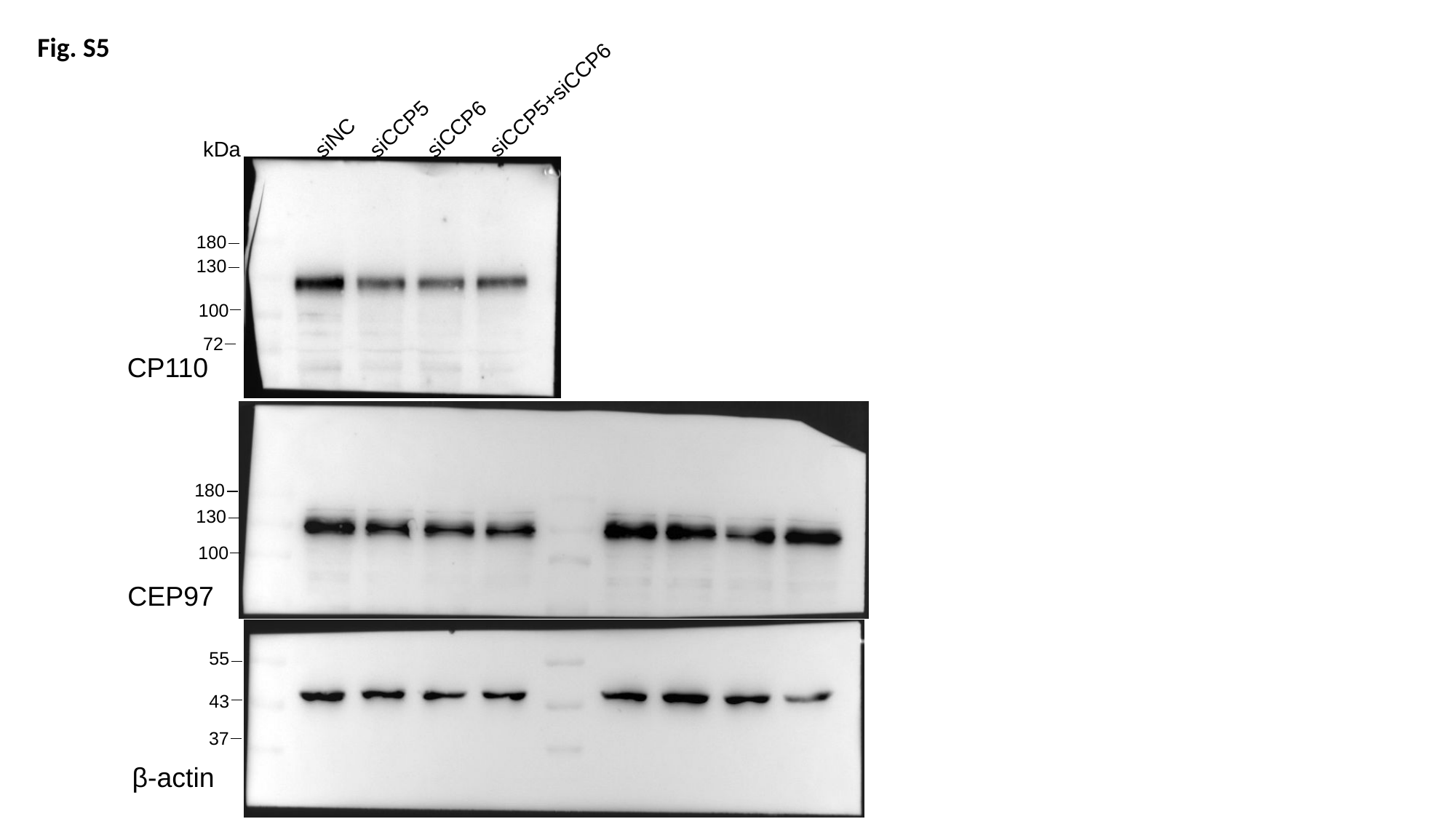

Fig. S5
siCCP5+siCCP6
siCCP5
siCCP6
siNC
kDa
180
130
100
72
CP110
180
130
100
CEP97
55
43
37
β-actin

## Slide 5
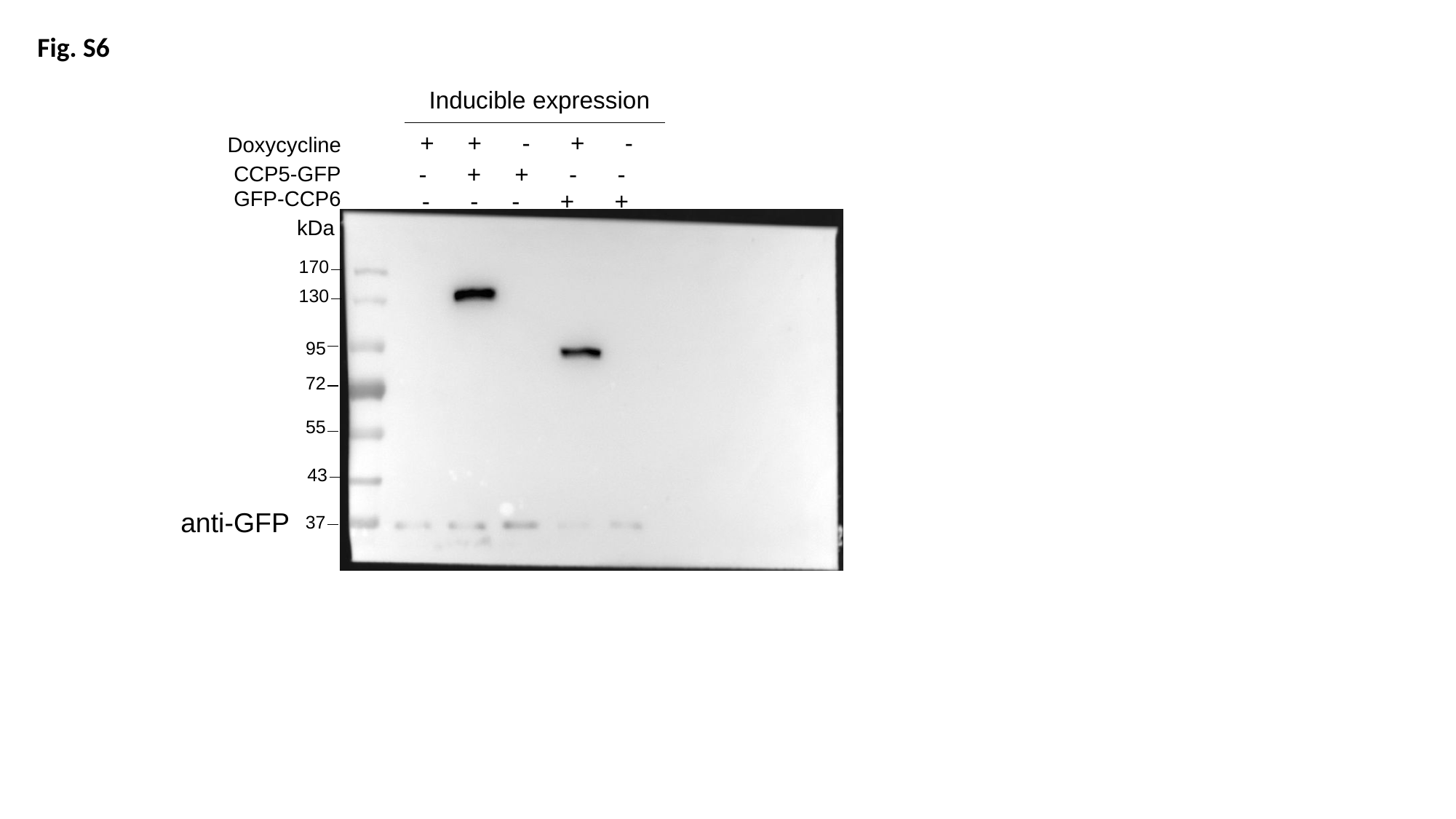

Fig. S6
Inducible expression
+ + - + -
Doxycycline
- + + - -
CCP5-GFP
GFP-CCP6
- - - + +
kDa
170
130
95
72
55
43
anti-GFP
37
